# Matrix stiffness maintains bladder cancer stemness via integrin-nuclear skeleton axis

**DOI:** 10.1038/s41419-025-08222-7

**Published:** 2025-12-12

**Authors:** Yiran Tao, Jiayu Huang, Jianfeng Hou, Zhuoer Hu, Tianyou Zhang, Zijun Mo, Kaixuan Zeng, Jialin Wu, Dejuan Wang, Jianguang Qiu

**Affiliations:** 1 Department of Urology, The Sixth Affiliated Hospital, Sun Yat-sen University, Guangzhou, Guangdong China; 2 Biomedical lnnovation Center, The Sixth Affiliated Hospital, Sun Yat-sen University, Guangzhou, Guangdong China; 3 Scientific Research Center, The Seventh Affiliated Hospital of Sun Yat-sen University, Shenzhen, Guangdong China; 4 Department of Orthopedic Surgery, The Seventh Affiliated Hospital of Sun Yat-sen University, Shenzhen, Guangdong China; 5 Center for Stem Cell Biology and Tissue Engineering, Key Laboratory for Stem Cells and Tissue Engineering, Ministry of Education, Sun Yat-Sen University, Guangzhou, Guangdong China; 6 National-Local Joint Engineering Research Center for Stem Cells and Regenerative Medicine, Zhongshan School of Medicine, Sun Yat-sen University, Guangzhou, Guangdong China; 7 School of Medicine, Sun Yat-sen University, Shenzhen, Guangdong China

**Keywords:** Cancer microenvironment, Bladder cancer

## Abstract

Tumors have a unique niche system that plays an important role in their occurrence and development. At present, there is increasing interest in the biomechanical properties of niches. The increased stemness of cancer cells is closely related to bladder cancer progression and recurrence. However, how biomechanical properties in the niche regulate bladder cancer stemness remains unclear. Here, we show that as bladder cancer progresses, matrix stiffness increases, and tumor stemness increases. Mechanistically, high matrix stiffness mediates β-catenin nuclear translocation by increasing the nuclear pore size. On the other hand, it promotes the expression of the nuclear cytoskeletal protein Lamin A/C, inhibits the nuclear export of β-catenin, and finally, it upregulates the Wnt pathway to increase the stemness of cancer cells. These findings reveal a role for matrix stiffness in the regulation of stemness in bladder cancer cells and suggest that targeting matrix stiffness may be an effective strategy to delay bladder cancer progression.

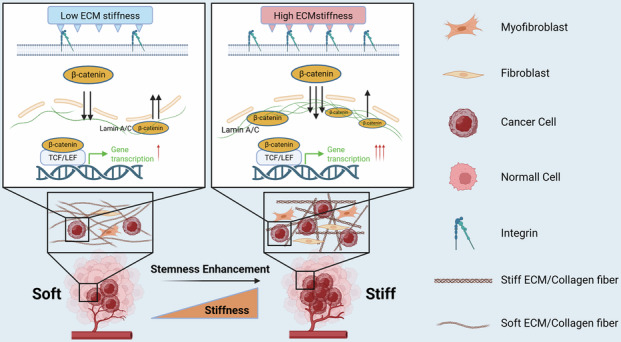

## Introduction

Urothelial carcinoma of the bladder, more commonly described as bladder cancer (BC), is the 10th most common malignancy worldwide. With high occurrence and mortality rates, it accounts for approximately 500,000 new cases and 200,000 deaths worldwide [1]. Approximately 75% of BC cases are classified as nonmuscle-invasive bladder cancer (NMIBC) on the basis of tumor depth, whereas the rest are termed muscle-invasive bladder cancer (MIBC) [2]. The primary standard treatments are surgery and platinum-based chemotherapy; current practices often cannot eradicate cancer, and patients suffer from high recurrence rates as well as metastasis [3]. Therefore, further attention is needed to address this critical issue.

Studies have revealed that cancer stem cells (CSCs) are a major cause of progressive malignancy in BC and other cancers [4]. In general, CSCs can self-renew and assume pivotal roles in tumor initiation and drug resistance via a myriad of mechanisms, such as paracrine transduction, epithelial‒mesenchymal transition and immunosuppression [5–7]. Bladder cancer stem cells (BCSCs), often derived from bladder epithelial stem cells or bladder cancer nonstem cells, exhibit rich clonal homogeneity and mainly express the surface markers CD44, 67LR, and BCMab1 [8, 9]. Although the stemness of BCSCs is known to be regulated by the tumor microenvironment, the underlying mechanism awaits investigation.

The tumor niche primarily refers to the noncancerous cells and components present in the tumor, including various immune cell types, cancer-associated fibroblasts, additional tissue-resident cell types and molecules constituting the extracellular matrix (ECM) [10]. Many studies have substantiated the diverse biological properties of the ECM, such as facilitating intercellular communication by acting as a reservoir for the sequestration of secreted molecules and as a substrate for cell adhesion and migration in the niche [11]. Recently, greater emphasis has been placed on the physical properties of the ECM, with matrix stiffness being one of the most important. Increased tissue stiffness is widely recognized as a mechanical abnormality in tumors and has been used as a diagnostic marker and prognostic factor [12]. ECM stiffness is related to tumor stemness and can regulate the self-renewal, proliferation, invasion and migration of cancer cells [13–15]. However, how the mechanical transmission of mechanical signals activates downstream stemness signaling pathways is still unclear.

Prominent signaling pathways, such as the Wnt, Notch and Hedgehog pathways, are closely connected with stemness maintenance and stem cell plasticity regulation in cancer [16]. Specifically, Wnt signaling consists of the extracellular ligand Wnts; trans-membrane receptors; intracellular compounds, including Dvl; a degradation complex comprising Gsk3β, CK1α, and Axin/conductin; and APC, β-catenin, and the transcription factor TCF/LEF [17]. Common cancer-associated Wnt hyperactivation can involve ligands that are reliant on or located downstream of the receptor interface. As evidenced by many studies, alterations in APC and Axin1 could disrupt the negative regulation of β-catenin by the destruction complex, whereas direct mutations of β-catenin could promote signaling by rendering itself insensitive to degradation [18]. However, with respect to mechanotransduction, few studies have explored the unique role of matrix stiffness in Wnt regulation in cancers.

Herein, we report that ECM stiffness can increase the stemness of BCSCs via the Wnt/β-catenin signaling pathway. Mechanistically, elevated matrix stiffness could facilitate the import of β-catenin by enlarging the nuclear core complex and by impacting nuclear Lamin A/C. Moreover, we also revealed the clinical significance of high ECM stiffness in bladder malignancies, offering novel insight into bladder cancer.

## Results

### Matrix stiffness and cancer cell stemness are increased during the progression of bladder cancer

**Fig. 1:**
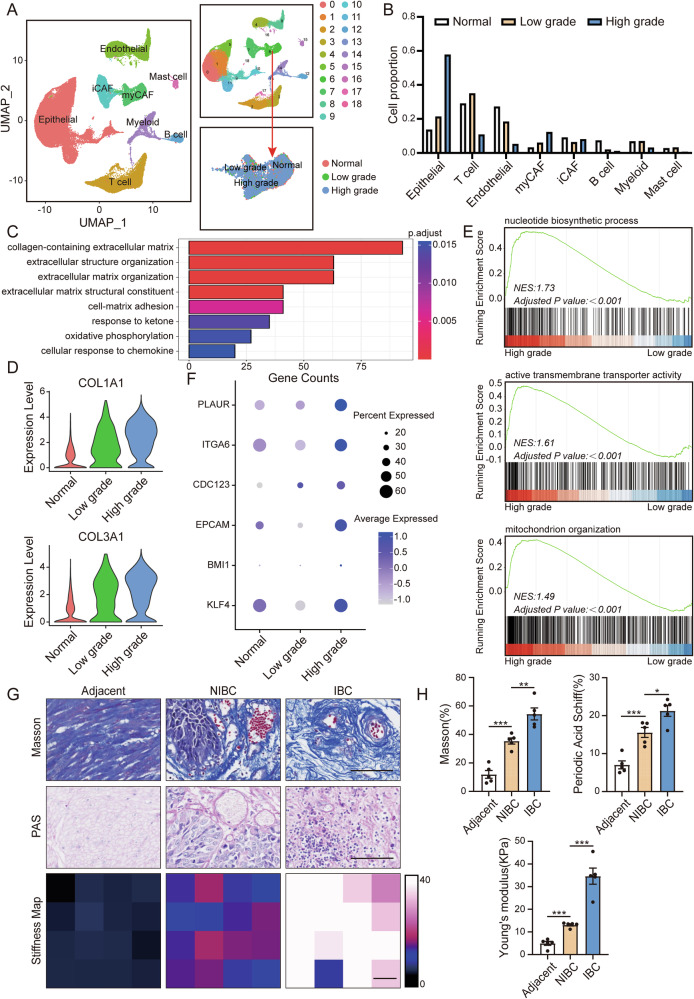
Matrix stiffness and cancer cell stemness are increased during the progression of bladder cancer. **A** Cell clusters of the sc-RNA-seq data from Chen et al. in figshare (https://figshare.com/articles/dataset/filtered_zip/23834670). **B** The histogram shows the proportions of various cells in normal, low-grade bladder cancer and high-grade bladder cancer samples. **C** The histogram shows that the differentially expressed genes of myCAFs in the low-grade group and high-grade group were significantly enriched in the gene sets. **D** GSEA of the hallmark gene sets in the MSigDB database revealing the enrichment of nucleotide biosynthetic process, active transmembrane transporter activity andochondrion organization GO terms in bladder cancer cells. NES, normalized enrichment score. **E** Expression levels of genes involved in extracellular matrix secretion. **F** Bubble plot showing the DEGs in bladder cancer cells in the aging group. **G** Representative images of Masson staining of tumor-adjacent, nonmuscle invasive bladder cancer (NIBC), and muscle invasive bladder cancer (IBC) samples. (bar = 100 μm). (n = 5 biological replicates for each group; unpaired t test). Representative images of PAS staining of adjacent, NIBC, and IBC samples. (bar = 100 μm). (n = 5 biological replicates for each group; unpaired t test). Thermal map of the matrix stiffness at the adjacent, NIBC, and IBC sites. (scale bar = 700 μm). (n = 5 biological replicates for each group; unpaired t test). **H** Quantitative statistical graph of (**G**). Data are represented as mean ± SEM. In all bar graphs, each dot represents one biological replicate. *p < 0.05, **p < 0.01, ***p < 0.001, ns no significance by one-way ANOVA (**H**).

To determine the intrinsic causes of bladder cancer progression, we conducted an in-depth analysis of the pathophysiological changes occurring in BC niches of different grades by analyzing the single-cell sequencing data of Chen et al. (Fig. 1A and Supplementary Fig. 1A) [19]. Compared with that in other nontumor cells, the proportion of tumor-associated fibroblasts in the high-grade BC niche was significantly greater. Among them, the increase was mainly in myCAF, while the change in iCAF was not significant, and there was only a slight decrease (Fig. 1B). Therefore, we performed gene set enrichment analysis on tumor-associated fibroblasts in low-grade and high-grade BC. Compared with those in low-grade BC, tumor-associated fibroblasts in high-grade BC presented an enrichment of genes related to extracellular matrix remodeling (Fig. 1C). Furthermore, an analysis of the genes differentially expressed between the two groups revealed that the expression levels of genes related to extracellular matrix secretion in the tumor-associated fibroblasts of high-grade BC patients were significantly greater (COL1A1, COL1A2, COL3A1, COL4A1, SPARC, DCN) (Fig. 1D and Supplementary Fig. 1B). The above results suggest that extracellular matrix deposition may be associated with the degree of malignant differentiation of BC.

To clarify the functional changes in BC cells, we subsequently performed gene set enrichment analysis on BC cells in low-grade and high-grade BC. We found that genes related to stemness were enriched in high-grade BC cells (Fig. 1E and Supplementary Fig. 1C). Through differential expression analysis of stemness-related genes in the two groups, we discovered that, compared with those in low-grade BC cells, the expression levels of stemness-related genes in high-grade BC cells were significantly greater (Fig. 1F). These results suggest that extracellular matrix deposition may promote the stemness of BC cells.

The deposition of ECM ultimately leads to alterations in the physical properties of the tumor, manifested as an increase in matrix stiffness. Additionally, during tumor progression, the enhanced stemness of tumor cells is a major factor. Therefore, we hypothesized that in the progression of BC, matrix stiffness may be involved in the regulation of tumor cell stemness. To verify this hypothesis, we collected adjacent, noninvasive bladder cancer (NIBC), and invasive bladder cancer (IBC) tissues. Some of the tissues were made into paraffin sections. Through Masson and PAS staining, we found that with the progression of BC, the number of collagen fibers and glycogen in the BC niche gradually increased. Another part of the tissues was made into frozen sections, and the stiffness of the BC tissues at different stages was measured via atomic force microscopy. The results revealed that with the progression of BC, the stiffness of tumor tissues gradually increased (Fig. 1G, H). Moreover, through the TCGA database, we conducted a prognostic analysis of genes related to extracellular matrix secretion and found that patients with high expression of extracellular matrix-related genes had a poorer prognosis (Supplementary Fig. 1D). In addition, we performed immunohistochemistry experiments on BC tissues at different stages and found that with the progression of BC, the expression levels of tumor stemness-related markers (CD44, OCT4, SOX2, NANOG) gradually increased (Supplementary Fig. 2A, B).

### High matrix stiffness promotes the stemness of bladder cancer cells

**Fig. 2:**
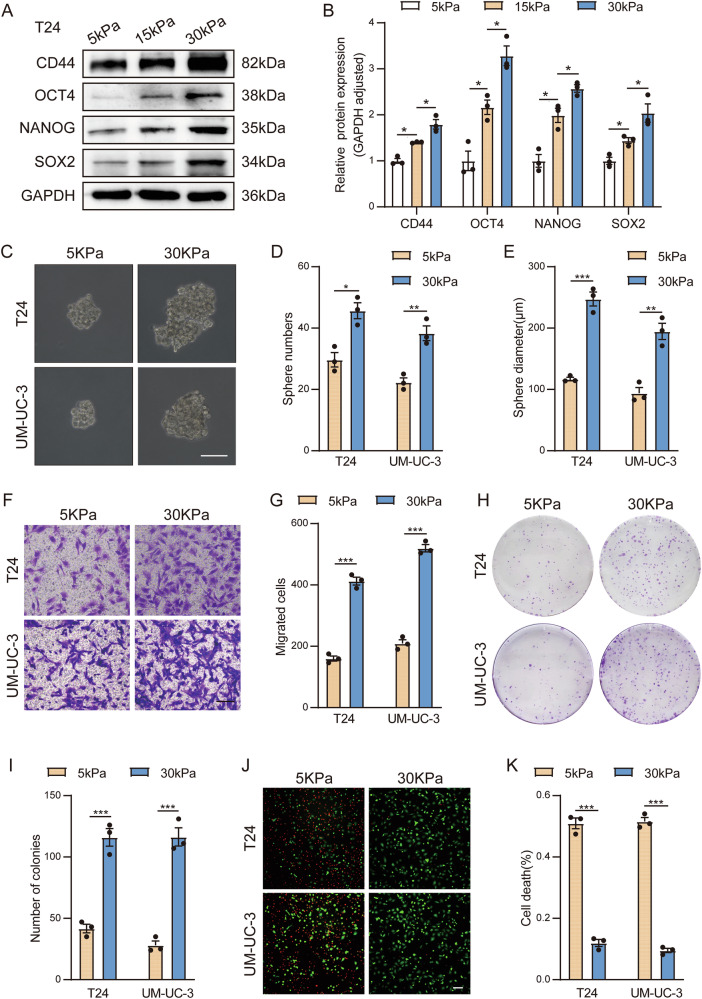
High matrix stiffness promotes the stemness of bladder cancer cells. **A**, **B** Western blot analysis and quantification of stemness marker expression in T24 cells cultured on 5 kPa, 16 kPa and 30 kPa polyacrylamide gels. **C** Spheres of representative images of the indicated T24 and UM-UC-3 cells cultured on 5 kPa and 30 kPa polyacrylamide gels. (bar = 100 μm). **D**, **E** Histograms showing the mean numbers and diameters of cultured spheres. **F** Transwell Matrigel invasion assay of representative images of the indicated T24 and UM-UC-3 cells cultured on 5 kPa and 30 kPa polyacrylamide gels. (bar = 200 μm). **G** Quantitative analyses of cell invasion through Matrigel-coated membranes. **H** Colony formation assay of representative images of the indicated T24 and UM-UC-3 cells cultured on 5 kPa and 30 kPa polyacrylamide gels. **I** Quantitative analyses of the number of cell colonies. **J** Calcein-AM/PI staining of representative images of the indicated T24 and UM-UC-3 cells cultured on 5 kPa and 30 kPa polyacrylamide gels. (survival, green + ; dead, red + ). (bar = 100 μm). **K** Quantitative statistical graph of (J). Data are represented as mean ± SEM. In all bar graphs, each dot represents one biological replicate. *p < 0.05, **p < 0.01, ***p < 0.001, ns no significance by unpaired Student’s *t* test (**D**, **E**, **G**, **I**, **K**) or one-way ANOVA (**B**).

To further confirm the impact of matrix stiffness on the stemness of BC cells, we first constructed polyacrylamide gels with stiffness levels of 5 kPa and 30 kPa in vitro. We subsequently utilized two BC cell lines, T24 and UM-UC-3, where T24 served as the primary experimental cell line and UM-UC-3 was used for partial experimental verification. We cultured BC cells on both soft and stiff polyacrylamide gels and then detected the expression of stemness-related markers at the protein level 48 h later. The results revealed that as stiffness increased, the expression of stemness markers in BC cells gradually increased (Fig. 2A, B and Supplementary Fig. 3A, B). After that, we carried out a series of phenotype experiments related to stemness. On the one hand, the results of the clonal sphere formation assay (Fig. 2C‒E), transwell assay (Fig. 2F, G), and colony formation assay (Fig. 2H, I) demonstrated that, compared with the BC cells cultured on soft polyacrylamide gels, those cultured on stiff polyacrylamide gels presented greater self-renewal, invasion, migration, and proliferation abilities. On the other hand, the enhanced drug resistance of tumor cells is a major manifestation of increased stemness. We applied cisplatin (DDP), a first-line chemotherapy drug for BC, and detected and calculated the IC50 values of the two cell lines through the CCK8 assay (Supplementary Fig. 3C, D). The results of the calcein/PI staining experiment revealed that the number of red-stained dead BC cells on stiff polyacrylamide gels was significantly reduced (Fig. 2J, K), indicating that tumor resistance to cisplatin was increased (Supplementary Fig. 3E, F). In summary, these results suggest that increased matrix stiffness during the progression of BC promotes the stemness of BC cells.

### Matrix stiffness promotes bladder cancer cell stemness by activating the Wnt pathway

**Fig. 3:**
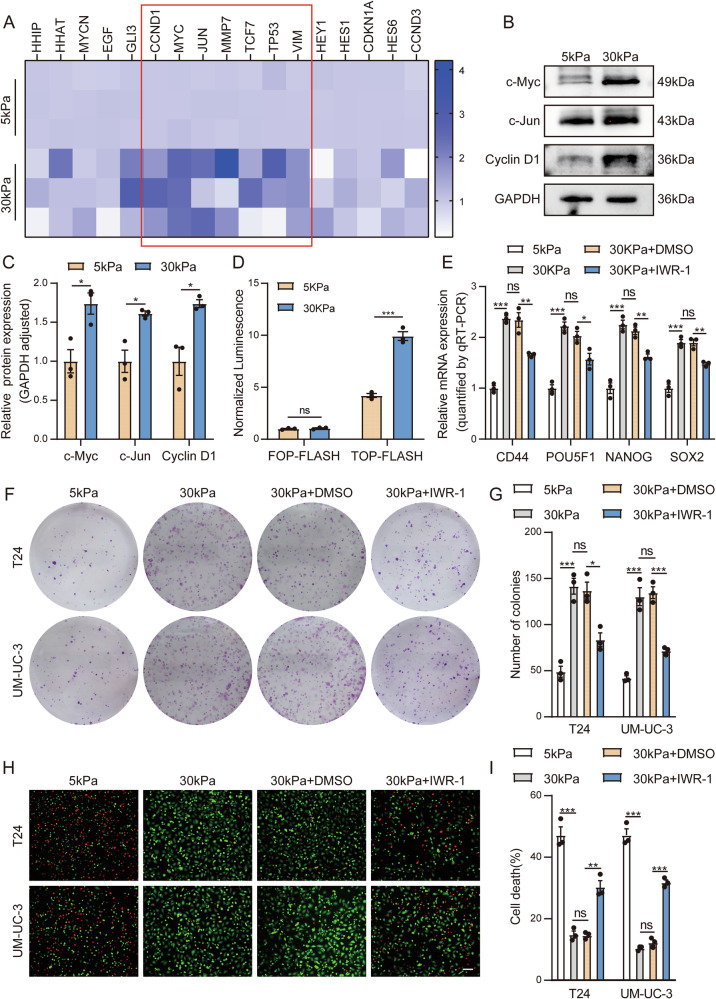
Matrix stiffness promotes bladder cancer cell stemness by activating the Wnt pathway. **A** qPCR analysis of the relative mRNA expression of Hedgehog, Wnt and Notch pathway downstream genes in T24 cells cultured on 5 kPa and 30 kPa polyacrylamide gels. **B**, **C** Western blot analysis and quantification of Wnt pathway downstream gene expression in T24 cells cultured on 5 kPa and 30 kPa polyacrylamide gels. **D** TOPFlash/FOP Flash luciferase reporter assays were conducted in the indicated T24 cells to measure TCF/LEF transcriptional activity. **E** qPCR analysis of the relative mRNA expression of stemness markers in T24 or T24 cells treated with IWR-1 cultured on 5 kPa and 30 kPa polyacrylamide gels. **F** Colony formation assay of representative images of the indicated T24 and UM-UC-3 cells treated with IWR-1 cultured on 5 kPa and 30 kPa polyacrylamide gels. **G** Quantitative analyses of the number of cell colonies. **H** Calcein-AM/PI staining of representative images of the indicated T24 and UM-UC-3 cells treated with IWR-1 cultured on 5 kPa and 30 kPa polyacrylamide gels. (survival, green + ; dead, red + ). (bar = 100 μm). **I** Quantitative statistical graph of (**H**). Data are represented as mean ± SEM. In all bar graphs, each dot represents one biological replicate. *p < 0.05, **p < 0.01, ***p < 0.001, ns, no significance by unpaired Student’s *t* test (**C**, **D**) or one-way ANOVA (**E**, **G,** **I**).

Studies have shown that the Hedgehog, Notch, and Wnt pathways play important roles in the regulation of bladder cancer cell stemness [20–22]. To further explore which stemness-related pathway is involved in the regulation of bladder cancer cell stemness by matrix stiffness, we detected the expression levels of genes downstream of these three pathways. We found that the expression of Wnt downstream genes on stiff polyacrylamide gels increased significantly (Fig. 3A and Supplementary Fig. 4A). The detection of the expression of genes downstream of the Wnt pathway at the protein level also yielded results consistent with the above findings (Fig. 3B, C). Moreover, the results of the luciferase reporter assay verified that the transcriptional activity level of TCF/LEF in the Wnt pathway increased in high-stiffness polyacrylamide gels (Fig. 3D).

To further verify the importance of the Wnt pathway in the regulation of bladder cell stemness by matrix stiffness, we applied the Wnt pathway inhibitor IWR-1. Through transwell assays, colony formation assays on plates, and Calcein-AM/PI live‒dead cell staining experiments, we detected changes in the stemness phenotypes of BC cells. We found that after the application of IWR-1, the expression of stemness markers in BC cells subjected to stiff polyacrylamide gels was significantly decreased (Fig. 3E and Supplementary Fig. 4B, C), and the invasion and migration abilities (Supplementary Fig. 4D, E), proliferation (Fig. 3F, G), and self-renewal abilities were obviously reduced (Supplementary Fig. 4F‒H). Moreover, the resistance of BC cells to cisplatin under stiff polyacrylamide gels was weakened (Supplementary Fig. 4I), and cell death increased (Fig. 3H, I). In addition, we applied SiRNA to interfere with the expression of β-catenin. The results showed that the expression of bladder cancer cell stemness-related genes decreased under high matrix stiffness after the application of SiRNA (Supplementary Fig. 5A). At the same time, the self-renewal, proliferation and drug resistance of tumor cells were significantly inhibited (Supplementary Fig. 5B–F). The above results indicate that high matrix stiffness enhances cell stemness by increasing the activation level of the Wnt pathway in BC cells, thereby improving their invasion, migration, proliferation, self-renewal, and drug resistance abilities.

### Matrix stiffness drives β-catenin nuclear translocation by increasing the nuclear pore size

**Fig. 4:**
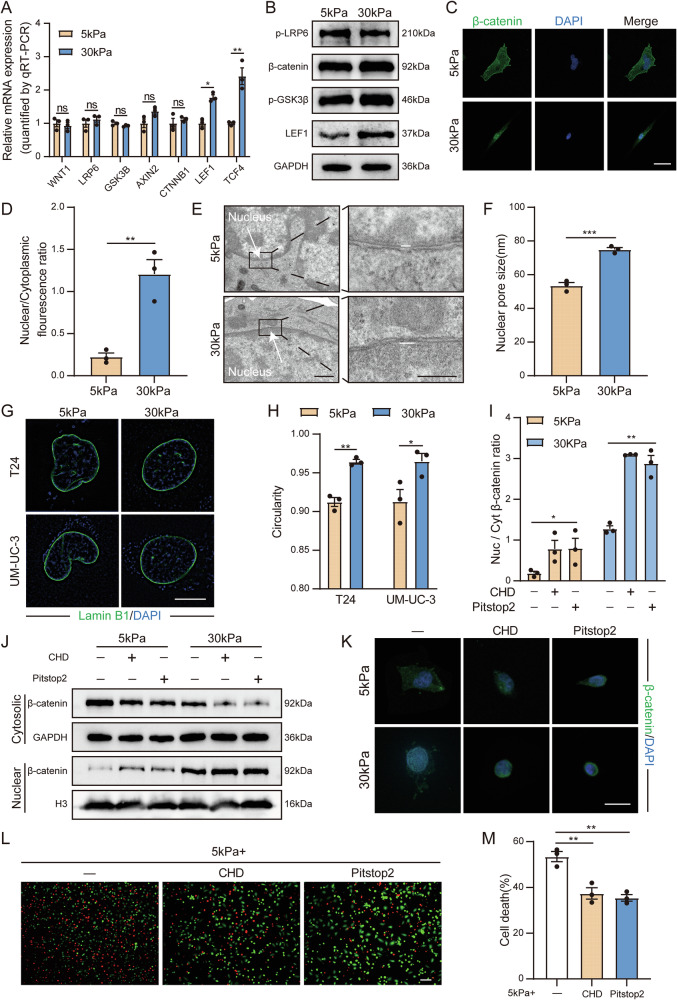
Matrix stiffness drives β-catenin nuclear translocation by increasing the nuclear pore size. **A** qPCR analysis of the relative mRNA expression of Wnt pathway-related genes in T24 cells cultured on 5 kPa and 30 kPa polyacrylamide gels. **B** Western blot analysis of Wnt pathway-related gene expression in T24 cells cultured on 5 kPa and 30 kPa polyacrylamide gels. **C** Representative immunofluorescence images of β-catenin in T24 cells cultured on 5 kPa and 30 kPa polyacrylamide gels. (bar = 50 μm). **D** Quantitative analysis of the β-catenin nuclear/cytoplasmic fluorescence ratio in (**C**). **E** Representative transmission electron microscopy (TEM) images of T24 cells cultured on 5 kPa and 30 kPa polyacrylamide gels. (left: bar = 500 nm, right: bar = 200 nm). **F** Quantitative analysis of the nuclear pore size in (**E**). **G** Representative immunofluorescence images of the nuclear membranes of T24 and UM-UC-3 cells cultured on 5 kPa and 30 kPa polyacrylamide gels. (bar = 10 μm). **H** Quantitative analysis of circularity in (**G**). **I**, **J** Western blot analysis and quantification of β-catenin expression in the nucleus and cytoplasm of T24 cells treated with CHD or Pitstop2 cultured on 5 kPa or 30 kPa polyacrylamide gels. **K** Representative immunofluorescence images of β-catenin in T24 cells treated with CHD and Pitstop2 cultured on 5 kPa and 30 kPa polyacrylamide gels. (bar = 50 μm). **L** Calcein-AM/PI staining of representative images of the indicated T24 cells treated with CHD and Pitstop2 cultured on 5 kPa polyacrylamide gels. (survival, green + ; dead, red + ). (bar = 100 μm). **M** Quantitative statistical graph of (**L**). Data are represented as mean ± SEM. In all bar graphs, each dot represents one biological replicate. *p < 0.05, **p < 0.01, ***p < 0.001, ns no significance by unpaired Student’s *t* test (**A**, **D**, **F**, **H**) or one-way ANOVA (**I**, **M**).

On the basis of the above results, we discovered that matrix stiffness regulates the stemness of BC cells via the Wnt pathway and subsequently delved deeper into which specific aspect of the Wnt pathway is influenced by matrix stiffness. Through qPCR and Western blotting, we found that there were no significant differences in the expression levels of the receptors of the Wnt pathway, the activation of the degradation complex, or the expression of β-catenin when exposed to polyacrylamide gels of different stiffness levels; however, the expression level of the TCF/LEF transcription elements within the nucleus increased significantly in the stiff polyacrylamide gels (Fig. 4A, B and Supplementary Fig. 6A). Research reports indicate that when the Wnt pathway is activated, β-catenin enters the nucleus, binds to LEF1 in the chromosomal region, and transactivates LEF1, and the increase in LEF1 further leads to the stable accumulation of β-catenin within the nucleus, establishing a positive feedback loop in the process of LEF1 expression and the recruitment of β-catenin into the nucleus [23, 24]. Therefore, we hypothesized that high matrix stiffness might upregulate the Wnt pathway by promoting the nuclear translocation of β-catenin, thereby increasing the expression level of TCF/LEF transcription elements through positive feedback. Through immunofluorescence imaging, we found that the nuclear‒cytoplasmic ratio of β-catenin increased in the presence of stiff polyacrylamide gels (Fig. 4C, D), further validating our hypothesis. The nuclear membrane is the “final checkpoint” for cells to sense mechanical stimuli and regulates the nuclear translocation of β-catenin [25]. We examined the nuclear pores of BC cells in polyacrylamide gels with different stiffnesses via transmission electron microscopy, and the results revealed that the size of the nuclear pores on the stiff polyacrylamide gels increased (Fig. 4E, F and Supplementary Fig. 6B, C), suggesting that high matrix stiffness promotes the nuclear translocation of β-catenin by increasing the size of the nuclear pores. Moreover, we observed changes in the morphology of the nuclear membrane via transmission electron microscopy (TEM) and subsequently observed the morphology of the nuclear membrane via superresolution imaging. Compared with those on soft polyacrylamide gels, the nuclear membrane on stiff polyacrylamide gels presented more “taut” (Fig. 4G, H).

Second, we perturbed nuclear pore permeability by disrupting FG interactions with trans1-2 cyclohexanediol (CHD) [26] and Pitstop2 [27], increasing the “effective open area” of the nuclear pores. The results confirmed that under soft polyacrylamide gels, part of the β-catenin originally located in the cytoplasm of the cells was transferred to the nucleus, and the amount of β-catenin in the nucleus further increased under stiff polyacrylamide gels (Fig. 4I‒K and Supplementary Fig. 6D, E). Moreover, after the application of CHD and Pitstop2, the expression of stemness markers in BC cells cultured on soft polyacrylamide gels increased (Supplementary Fig. 6F). The resistance of BC cells to cisplatin increased (Fig. 4L, M and Supplementary Fig. 6G, H), and their self-renewal ability was enhanced (Supplementary Fig. 6I, J). The above results indicate that nuclear pores are involved in the regulation of the Wnt pathway in BC cells by matrix stiffness.

Subsequently, we delved into the upstream region of the nuclear skeleton. The research indicates that focal adhesions are crucial mediators for the transmission of biomechanical signals within cells, and integrins are the key components among them [28]. Our detection at the mRNA and protein levels revealed that in high matrix stiffness conditions, the expression level of integrins in bladder cancer cells was higher, and the cytoskeleton underwent remodeling (Supplementary Fig. 7A–C). At the same time, FAK/Src pathway downstream of integrins was upregulated. By applying cilengitide (an integrin receptor inhibitor) to inhibit the transmission of mechanical signals, the content of active β-catenin in bladder cancer cells under stiff polyacrylamide gels could be reduced (Supplementary Fig. 7D, E). Biomechanical signals are transmitted through focal adhesions on the cell membrane to the cytoskeleton, and finally reach the cell nucleus. The LINC complex is an important mediator for transmitting biomechanical signals to the nucleus [29–31]. Subsequently, we conducted detection on the various components of the LINC complex and found that the expressions of SUN1, SUN2, SYNE2, and SYNE3 were elevated in high matrix stiffness conditions, with SYNE2 being the most significant (Supplementary Fig. 7F). Furthermore, we used siRNA to interfere with the expression of Nesprin2 (the product of SYNE2 gene). The results showed that both Si Nesprin2 and cilengitide could reduce the nuclear aggregation of β-catenin under high matrix stiffness (Supplementary Fig. 7G, H). Ultimately, it leads to the down-regulation of genes related to stemness (Supplementary Fig. 7I). These results indicate that the integrin-LINC complex mediates the transmission of biomechanical signals to the cell nucleus.

### The nuclear skeleton protein Lamin A/C binds β-catenin to inhibit its nuclear exit

**Fig. 5:**
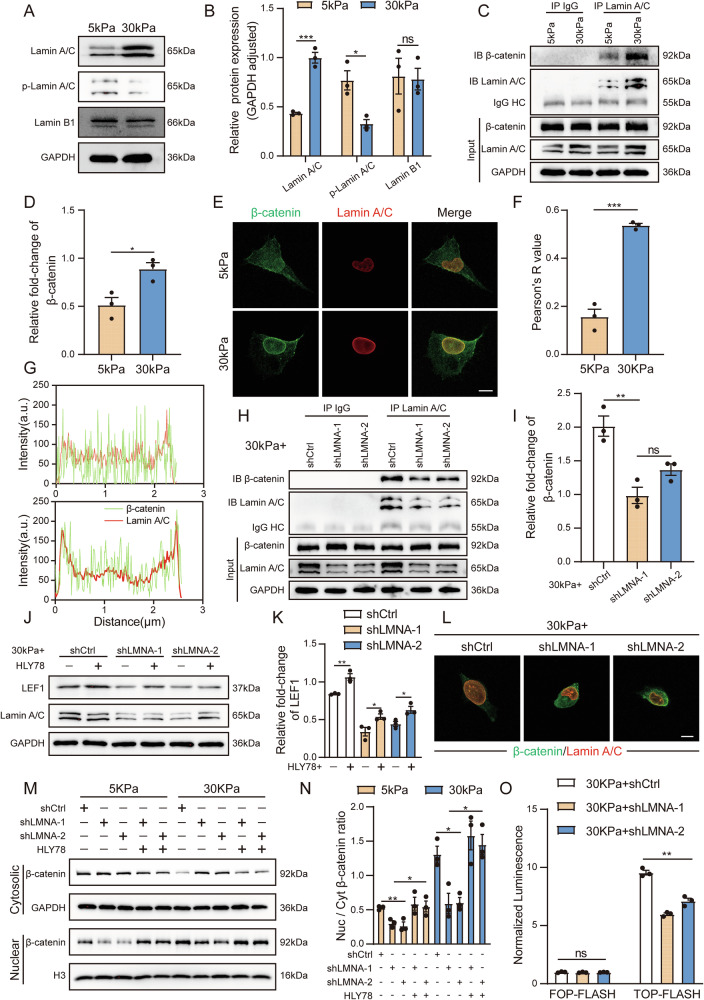
The nuclear skeleton protein Lamin A/C binds β-catenin to inhibit its nuclear exit. **A**, **B** Western blot analysis and quantification of lamin expression in T24 cells cultured on 5 kPa and 30 kPa polyacrylamide gels. **C** T24 cells were cultured on 5 kPa and 30 kPa polyacrylamide gels. The lysates were incubated with an anti-Lamin A/C antibody for 12 h and then conjugated with agarose. Bound proteins were analyzed by immunoblotting with an anti-β-catenin antibody. Input samples were taken prior to immunoprecipitation and immunoblotted with the indicated antibodies. **D** Quantitative statistical graph of (**C**). **E** Representative immunofluorescence images of β-catenin and Lamin A/C in T24 cells cultured on 5 kPa and 30 kPa polyacrylamide gels. (bar = 10 μm). **F** Colocalization analysis of the fluorescence intensity in (**E**). **G** Quantitative statistical graph of (**F**). **H**, **I** Co-IP assay analysis and quantification of β-catenin expression in T24 cells with or without Lamin A/C depletion cultured on 30 kPa polyacrylamide gels. **J**, **K** Western blot analysis and quantification of LEF1 and Lamin A/C expression in T24 cells with or without Lamin A/C depletion cultured on 30 kPa polyacrylamide gels. **L** Representative immunofluorescence images of β-catenin and Lamin A/C in T24 cells with or without Lamin A/C depletion cultured on 30 kPa polyacrylamide gels. (bar = 10 μm). **M**, **N** Western blot analysis and quantification of β-catenin expression in the nucleus and cytoplasm of T24 cells with or without Lamin A/C depletion and HLY78 cells cultured on 5 kPa and 30 kPa polyacrylamide gels. **O** TOPFlash/FOPFlash luciferase reporter assays were conducted in the indicated T24 cells with or without Lamin A/C depletion to measure TCF/LEF transcriptional activity. Data are represented as mean ± SEM. In all bar graphs, each dot represents one biological replicate. *p < 0.05, **p < 0.01, ***p < 0.001, ns no significance by unpaired Student’s *t* test (**B**, **D**, **F**) or one-way ANOVA (**I**, **K**, **N**, **O**).

In the previous section, we observed that the nuclear membrane presented different morphologies under polyacrylamide gels of different stiffness levels. The morphology of the nuclear membrane is related to the nuclear lamins in the nuclear skeleton system [32]. Moreover, our previous experiments revealed that after β-catenin entered the nucleus in stiff polyacrylamide gels, it aggregated around the nuclear lamina. Therefore, we hypothesized that in addition to the structure of the nuclear skeleton, the components of the nuclear skeleton—the nuclear lamins—are also involved in the regulation of the Wnt pathway and might interact and bind with β-catenin. We subsequently detected nuclear lamins and found that among the nuclear lamins in the stiff polyacrylamide gels, the increase in Lamin A/C was the most significant, Lamin A/C degradation (p-Lamin A/C) decreased, Lamin B1 did not change significantly (Fig. 5A, B and Supplementary Fig. 8A, B). On the one hand, our Co-IP experiments demonstrated that the amount of Lamin A/C bound to β-catenin in BC cells cultured on stiff polyacrylamide gels was greater than that in the cells cultured on soft polyacrylamide gels (Fig. 5C, D and Supplementary Fig. 8C, D). On the other hand, through immunofluorescence colocalization analysis, we found that Lamin A/C and β-catenin were colocalized, and the Pearson correlation coefficient of their colocalization was greater in the stiff polyacrylamide gels (Fig. 5E‒G). These results indicate that there is an interaction and binding relationship between Lamin A/C and β-catenin.

To verify the role of Lamin A/C in regulating the Wnt pathway, we constructed BC cell lines with stable interference of Lamin A/C expression. The results of protein immunoblotting showed that after interference with Lamin A/C, the amount of Lamin A/C bound to β-catenin on stiff polyacrylamide gels decreased significantly. But the application of the Wnt pathway agonist HLY78 did not increase the expression of Lamin A/C (Fig. 5H‒K and Supplementary Fig. 8E, F). The above results indicate that the changes in the Wnt pathway do not in turn affect the expression of Lamin A/C. Meanwhile, the nuclear membrane morphology changes after being interfered with Lamin A/C (Fig. 5L). In terms of influencing the nuclear‒cytoplasmic distribution of β-catenin, after interference with Lamin A/C, the export of β-catenin from the nucleus increased, and the nuclear‒cytoplasmic ratio decreased (Fig. 5M, N and Supplementary Fig. 8G, H). Moreover, the results of the luciferase reporter assay revealed that the activation level of the Wnt pathway decreased (Fig. 5O and Supplementary Fig. 8I). Further detection of the drug resistance of BC cells revealed that after interference with Lamin A/C, the resistance of tumor cells on stiff polyacrylamide gels to cisplatin weakened (Supplementary Fig. 8J, K). The above results suggest that the regulation of the Wnt pathway in BC cells by matrix stiffness may be achieved through the joint action of nuclear pores and Lamin A/C.

### High matrix stiffness promotes tumor regrowth in mice

**Fig. 6:**
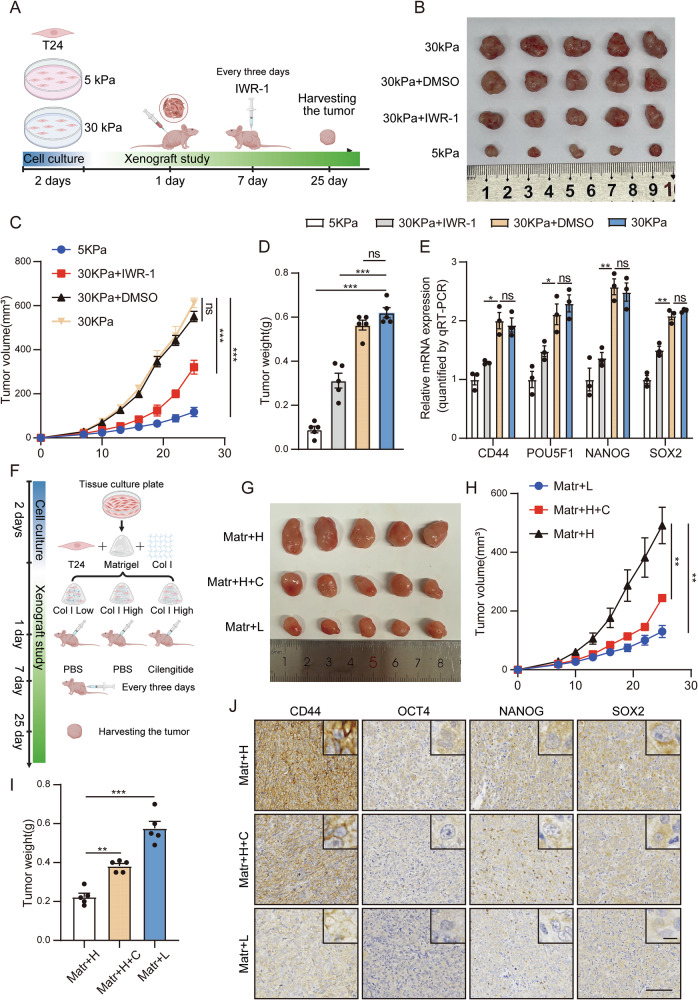
High matrix stiffness promotes tumor regrowth in mice. **A** Schematic representation of in vivo model I. T24 cells were seeded on 5 kPa and 30 kPa polyacrylamide gels. After 48 h, the cells were collected and injected into the subcutaneous tissue of the mice. **B** Representative images of xenograft tumors after IWR-1 treatment (n = 5). **C** Tumor volume was determined every 3 days after IWR-1 treatment. The IWR-1 treatment was given on the seventh day. **D** Tumor weights were measured after the tumors were isolated from the mice. **E** qPCR analysis of the relative mRNA expression of stemness markers in xenograft tumors. **F** Schematic representation of in vivo Model II. T24 cells without any treatment were cultured in dishes, digested and collected, resuspended in Matrigel, and then supplemented with different amounts of rat tail collagen I to create a growth microenvironment with different matrix stiffnesses. Finally, T24 cells were injected subcutaneously into the mice. **G** Representative images of xenograft tumors after cilengitide (**C**) treatment (n = 5). **H** Tumor volume was determined every 3 days after C treatment. The C treatment was given on the seventh day. **I** Tumor weights were measured after the tumors were isolated from the mice. **J** Immunohistochemical staining for CD44, OCT4, NANOG and SOX2. Magnification ×10 (lower, bar = 100 μm) and ×40 (upper, bar = 10 μm). Data are represented as mean ± SEM. In all bar graphs, each dot represents one biological replicate. *p < 0.05, **p < 0.01, ***p < 0.001, ns, no significance by one-way ANOVA (**C**–**E**, **H**, **I**).

Here, we further verified the impact of matrix stiffness on the stemness of BC cells through in vivo experiments. Studies have shown that tumor cells generate specific mechanical memory in response to mechanical signals, enabling them to retain their corresponding biological functions even after the mechanical signals disappear [33]. On this basis, we constructed the first in vivo model. First, we cultured T24 cells on 5 kPa and 30 kPa gels. After 48 hours, we collected the cells and injected them subcutaneously into the mice. Finally, we observed tumor growth (Fig. 6A). The results showed that the T24 cells cultured on stiff polyacrylamide gels had greater tumorigenic ability. The subcutaneous tumors grew faster and had a larger volume. Moreover, the subcutaneous injection of IWR-1 effectively weakened the tumorigenic ability of T24 cells and delayed the growth of subcutaneous tumors (Fig. 6B‒D). We further extracted mRNA and protein from the subcutaneous tumors. Through qPCR and Western blotting, we found that the expression levels of stemness markers and the activation level of the Wnt pathway in subcutaneous tumors at 30 kPa were significantly greater than those at 5 kPa. However, the application of IWR-1 effectively reduced stemness (Fig. 6E and Supplementary Fig. 9A‒C).

Because the cells cannot receive mechanical signal stimulation continuously in Model I, we designed the second model. First, we cultured untreated T24 cells in a culture dish. After digestion and collection, we resuspended the cells in Matrigel. Then, we added different amounts of rat tail collagen I, thereby constructing growth microenvironments with different matrix stiffness levels. Finally, we injected the mixture subcutaneously into the mice (Fig. 6F). Through observation, we found that high matrix stiffness promoted the growth of subcutaneous tumors. The application of cilengitide to target matrix stiffness effectively slowed the growth of subcutaneous tumors (Fig. 6G‒I). Furthermore, we detected the expression levels of stemness markers through immunohistochemistry and Western blotting. The results revealed that high matrix stiffness promoted the expression of stemness markers (Fig. 6J and Supplementary Fig. 9D, E). Moreover, the qPCR results indicated that, compared with those in the low matrix stiffness group, the expression levels of genes downstream of the Wnt pathway were greater in the high matrix stiffness group, and the above results could be significantly inhibited by cilengitide (Supplementary Fig. 9F). Overall, this study is the first to demonstrate that high matrix stiffness can mediate the activation of the Wnt pathway through the nuclear skeleton system in BC.

### Inhibiting the Wnt pathway and mechanotransduction alleviates the facilitating effect of high matrix stiffness on bladder cancer organoids

**Fig. 7:**
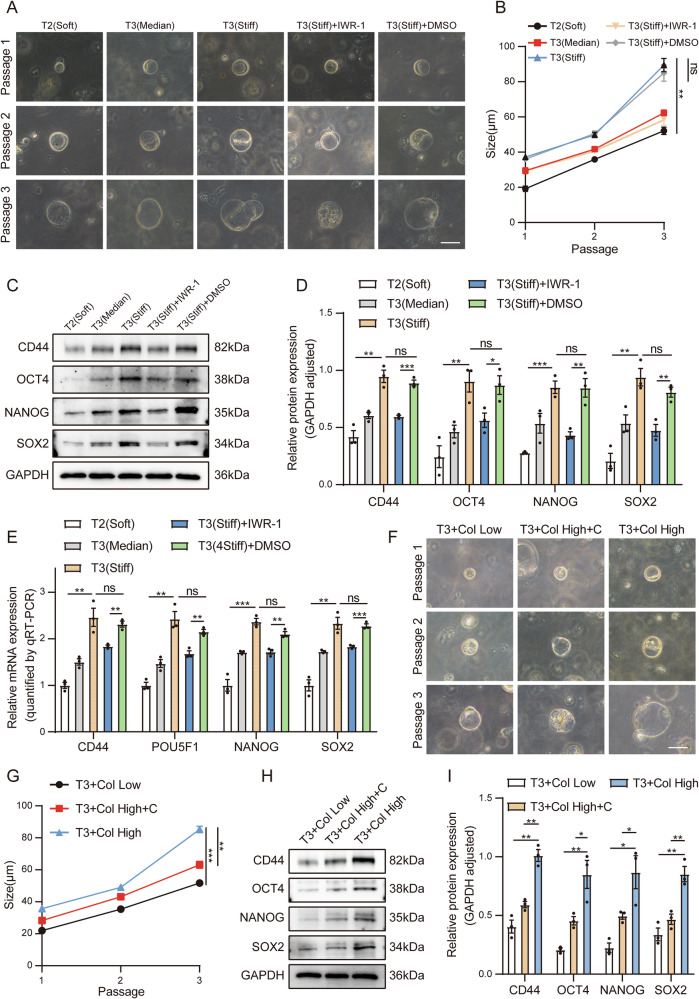
Inhibiting the Wnt pathway and mechanotransduction alleviates the facilitating effect of high matrix stiffness on bladder cancer organoids. **A** Images showing the typical morphology of organoids cultured with or without IWR-1 over three passages of organoid model I (bar = 50 μm). **B** Line chart showing the mean diameters of the organoids cultured in (**A**). **C**, **D** Western blot analysis and quantification of stemness marker expression in organoid Model I. **E** qPCR analysis of the relative mRNA expression of stemness markers in organoid Model I. **F** Images showing the typical morphology of organoids cultured with or without cilengitide (**C**) over three passages in organoid Model II. (bar = 50 μm). **G** Line chart showing the mean diameters of the organoids cultured in (**F**). **H**, **I** Western blot analysis and quantification of stemness marker expression in Model II organoids. Data are represented as mean ± SEM. In all bar graphs, each dot represents one biological replicate. *p < 0.05, **p < 0.01, ***p < 0.001, ns, no significance by one-way ANOVA (**B**, **D**, **E**, **G**, **I**).

Organoids are three-dimensional microorganisms cultivated in vitro. They possess a genetic background and histological characteristics highly similar to those in vivo. With a complex and comparable structure to real organs, they can also partially reproduce the source tissues and organs, making them an ideal research model [34]. In this study, we constructed a human BC organoid model. Analogous to the approach in the in vivo mouse experiments, in Model I, we took tissue samples from three BC patients (TMN staging: 1 patient in stage T2 and 2 patients in stage T3) for atomic force microscopy detection. The results revealed that the stiffness of these three BC tissues was approximately T2: 2 kPa (Soft), T3: 20 kPa (Median), and T3: 34 kPa (Stiff) (Supplementary Fig. 10A). We subsequently used the remaining tissues to construct BC organoids. We measured the diameter of each generation of organoids and plotted a growth curve. In terms of the diameter of the organoids, the T3 (Stiff) group > the T3 (median) group > the T2 (soft) group. The application of IWR-1 inhibited the growth of the organoids in the T3 (Stiff) group (Fig. 7A, B). When the organoids were cultured to the third generation, we extracted the mRNAs and proteins from the organoids in each group for detection. The results revealed that the Ki67 proliferation index was also high in the organoids cultured from high-stiffness BC tissues (Supplementary Fig. 10B). Moreover, the organoids cultured from BC tissues with high stiffness presented increased expression levels of stemness markers and genes downstream of the Wnt pathway (Fig. 7C‒E and Supplementary Fig. 10C).

In Model II, we also added different amounts of rat tail collagen I to the Matrigel in which the BC organoids grew. By plotting the growth curves, we found that high matrix stiffness promoted the growth of the organoids, and the application of cilengitide to target matrix stiffness effectively slowed the growth of the organoids (Fig. 7F, G). Further detection of the expression of stemness markers at the transcriptional and translational levels revealed that high matrix stiffness promoted the expression of stemness markers (Fig. 7H, I and Supplementary Fig. 10D). Moreover, the expression of genes downstream of the Wnt pathway and Ki67 also increased accordingly, and these effects were significantly inhibited by cilengitide (Supplementary Fig. 10E, F).

These findings indicate that high matrix stiffness promotes the stemness of BC cells. Importantly, the nuclear skeleton system plays an irreplaceable role in this process, possibly by jointly mediating the accumulation of β-catenin in the nucleus through nuclear pores and Lamin A/C (Graphical Abstract).

## Discussion

Although substantial research into the progression of BC has been carried out, the exact mechanism promoting cancer cell stemness and hindering related treatment remains to be elucidated. Here, we revealed the underlying pathway of the increase in BC cell stemness through the Wnt/β-catenin pathway upon high matrix stiffness. More specifically, increased matrix stiffness increased the opening of nuclear pore complexes (NPCs) and promoted the expression of Lamin A/C via mechanotransduction, which in turn aggravated the intranuclear β-catenin content and activated relevant transcription. Furthermore, increasing matrix stiffness could effectively decrease the stemness of BC cells and thus relieve the progression of BC.

The mechanical properties of the niche are known to include matrix stiffness, hydrostatic pressure, fluid shear stress and extracellular fluid viscosity, among which matrix stiffness is considered a major factor in solid tumors [35]. As evidenced by substantial research in breast cancer and other malignancies, matrix stiffness can increase the malignant phenotype of tumor and stromal cells by promoting proliferation and drug resistance through the Ras/MAPK cascade, epithelial‒mesenchymal transition and various other behaviors, such as M2 macrophage polarization [12, 13, 36]. The stemness of cancer cells is also closely correlated with matrix stiffness, and in this study, we clarified that increased matrix stiffness could also enhance the stemness of BC cells in both in vivo and in vitro experiments. Accordingly, increased levels of Ki67 and PCNA, as well as more HepG2 cells, were observed in the background of liver cirrhosis, revealing increased proliferation ability in a stiffer environment [37]. Cell proliferation and drug resistance ability was also found to be significantly increased when laryngeal cancer cells were cultured on stiffer substrates, as evidenced by the findings of Hui et al. and Robey et al., which was consistent with our findings [38, 39]. In contrast, opposite patterns could be observed in other types of cancer cells. In the case of osteosarcoma or glioblastoma CSCs, the stemness level is maintained when matrix stiffness is reduced, and a stiffer substrate contributes to the growth and metastasis of the tumor [40, 41]. This discrepancy might be related to the usual metastatic niche and peripheral edema [42]. However, the exact underlying mechanism has yet to be revealed.

The mechanical properties of the ECM regulate fundamental cellular behaviors through mechanotransduction, a process that includes mechanosensing and downstream events [43]. In mechanosensing, integrins act as key mechanical sensors on the cell surface and play a central role: via the heterodimeric structure formed by α/β subunits, they specifically recognize ligands such as collagen and fibronectin in the ECM [44, 45]. When tissue elasticity increases, the affinity between the ECM and integrins is significantly enhanced, triggering a conformational change in the receptor and converting it from a low-activity state to a high-activity state [46]. The cytoplasmic tail of activated integrins can recruit the adaptor protein talin, and directly bind to talin via the FERM domain. This interaction relieves talin’s intramolecular autoinhibitory conformation, exposing its actin-binding sites, which in turn initiates the polymerization and assembly of actin monomers to form a continuous microfilament network. These actin filaments further bind to myosin II, generating contractile forces through cross-bridge cycling, thereby forming stress fibers with mechanotransductive functions [47–49]. This “ECM-integrin-cytoskeleton” axis not only enhances the mechanical stability of cells through the fibrous network and maintains the morphological integrity of cells in the mechanical microenvironment, but also converts extracellular mechanical signals into intracellular biochemical signals. It provides initial mechanical stimuli for the subsequent activation of pathways such as YAP/TAZ and FAK/Src, enabling the transmembrane transmission from extracellular mechanical signals to intracellular biological effects [50–52]. Here, we applied single-cell sequencing to further confirm the abnormal deposition of extracellular matrix in invasive bladder cancer and, for the first time, revealed that high matrix stiffness is a crucial factor influencing the progression of bladder cancer. Under high matrix stiffness culture conditions, bladder cancer cells exhibit higher expression levels of integrins, accompanied by the upregulation of the FAK/Src pathway and cytoskeletal remodeling.

The transmission of mechanical signals activates a series of downstream transduction processes [53]. NPC and the nuclear lamina are key components of nuclear mechanotransduction. As emphasized by Elosegui-Artola et al., stiffer environments transmit forces from focal adhesions to the cell nucleus via the cytoskeleton, resulting in the stretching and opening of NPCs [54–57]; Our study also confirmed that when matrix stiffness increases, the NPC openings in BCs are enhanced and the nuclear membrane tension is augmented. Whether ECM stiffness directly induces such changes requires further investigation. Laminins are the main structural components of the nuclear lamina; they are dynamic proteins that assemble and disassemble in response to different stimuli, endowing the laminin framework with diverse mechanical properties [58]. Among various lamins, Lamin A/C plays a central role in mechanotransduction. Recent studies have investigated the link between matrix remodeling, nuclear Lamin A/C, and nuclear β-catenin in skeletal stem cells, indicating that Lamin A/C levels control the localization of emerin, and the binding of emerin to β-catenin regulates the transcription of target genes [59, 60]. Here, we report a direct interaction between Lamin A/C and β-catenin. In BCs cultured on stiffer substrates, both the expression of Lamin A/C and its binding to β-catenin are enhanced. Therefore, Lamin A/C could serve as a potent target for regulating the response of bladder cancer cells to matrix stiffness, thereby attenuating the stemness of tumor cells.

Among signaling cascades, the Wnt/β-catenin pathway has been widely recognized as a key regulator in cancer initiation, progression, and the maintenance of stemness [61]. A large body of existing research has demonstrated that aberrations in the Wnt/β-catenin pathway exert a significant impact on the initiation and progression of bladder cancer. For instance, BCL9L, as a co-activator of β-catenin, shows that its upregulated expression can markedly enhance the activity of the Wnt/β-catenin pathway in bladder cancer cells, thereby promoting their proliferation, migration, and invasion. Additionally, previous studies have mainly focused on the paracrine regulation of the Wnt/β-catenin pathway, such as through exosomes, lncRNAs, and microRNAs [62, 63]. Studies have reported that microRNA-135a can activate the Wnt/β-catenin signaling pathway by downregulating glycogen synthase kinase 3β (GSK-3β), thereby accelerating the EMT and migration of bladder cancer cells [64]. Our study reveals a novel mechanism that influences the nuclear/cytoplasmic distribution of β-catenin. On the other hand, the impact of biomechanical properties on cellular biological functions has attracted increasing attention. Among them, most existing evidence indicates that the Hippo/YAP pathway is affected by ECM stiffness [65–67]. Our study reveals the unique role of ECM stiffness in upregulating the Wnt/β-catenin pathway, thereby providing a novel perspective.

Given the crucial role of ECM stiffness in the progression of bladder cancer, detecting tumor hardness may contribute to tumor diagnosis, staging, classification, and predicting the prognosis of cancer patients [68, 69]. The biophysical effects of ECM stiffness on tumors may interfere with drug delivery and sensitivity to anticancer drugs [69]. Therefore, detecting tumor hardness may help in the stratified treatment of patients. Targeting integrins and Lamin A/C to block ECM rigidity-induced mechanotransduction is expected to inhibit the progression of bladder cancer. However, due to the complex role of ECM in tumor progression and the dynamic nature of ECM remodeling, there may be many obstacles and challenges in targeting ECM rigidity in tumors. As our knowledge of the biology of the tumor extracellular matrix expands, we look forward to identifying more targets and developing more promising antitumor therapeutic drugs.

Nevertheless, there are still limitations in our study. First, the in vitro culture substrate we employed was polyacrylamide, which cannot fully simulate the in vivo niche; therefore, more appropriate biomaterials are needed. Second, the experiments involved were performed in a two-dimensional landscape, so further verification in three dimensions should be carried out to mimic the in vivo environment. Finally, to better cope with comprehensive cancer treatment, other physical properties of the niche, such as hydrostatic pressure, fluid shear stress and extracellular fluid viscosity, should also be thoroughly investigated.

## Conclusion

Our study reveals that under high ECM stiffness, the stemness of BCs is upregulated through the Wnt/β-catenin pathway. More specifically, increased matrix stiffness enlarges the openings of NPCs and promotes the expression of lamin A/C via mechanotransduction. This, in turn, increases the nuclear β-catenin content and activates related transcription. Furthermore, targeting the stiffness of the TME can effectively reduce the stemness of BC cells, thereby alleviating the progression of BC. Therefore, this research will provide new insights into understanding bladder malignancy and lay a solid foundation for improving cancer treatment in the future.

## Materials and methods

### Ethics statement

All procedures involving human samples were performed with the approval of the internal review and ethics boards of the Sixth Affiliated Hospital of Sun Yat-sen University (Accreditation number: 2022ZSLYEC-620). The mouse experiments were carried out according to protocols approved by the Institutional Animal Care and Use Committee (ACUC) of Shenzhen Top Biotech. Co., Ltd. (approval number: TOPGM-IACUC-2023-0206). The mice were housed at Top Biotech. Co., Ltd. (Shenzhen, China) under specific pathogen-free (SPF) conditions at 23 ± 2 °C ambient temperature with 40% humidity and a 12 h light/dark cycle. In this study, the number of subcutaneous tumors in the mice did not exceed the maximum tumor size/burden.

### Patients and tissue specimens

In this study, human bladder cancer tissue samples were collected between 2022 and 2023 at the Sixth Affiliated Hospital of Sun Yat-sen University; 5 patients with nonmuscle invasive bladder cancer and 5 patients with muscle invasive bladder cancer were included. All patients have been informed and have given their consent for their samples to be included in this study.

### Cell culture, SiRNAs and stable cell lines

The human bladder cancer cell lines T24 and UM-UC-3 were obtained from Sun Yat-Sen Memorial Hospital and grown in RPMI 1640 medium (Gibco, USA) supplemented with 1% penicillin‒streptomycin and 10% fetal bovine serum at 37 °C with 5% CO_2_. Human β-catenin (CGGAGGAGAUGUACAUUCAdTdT) and Nesprin2 (GAUACUCGGAGACGCAUAUdTdT) siRNAs were purchased from RiboBio. For gene silencing, riboFECT CP Transfection Kit (RiboBio, Guangzhou, China) was used for transfection. Lentivirus packaging was supplied by Guangzhou RiboBio Co., Ltd. The lentiviral vector plasmid pLL3.7 (Addgene, MA, USA) was used to clone the following sense sequences to construct stable clones:

shLMNA-1: GCGCCAGAATGGAGATGAT;

shLMNA-2: GCACTGCTCTCAGTGAGAA.

We generated stable knockdown cells as previously described [70]. Briefly, lentiviral particles (multiplicity of infection: 30) were used to infect T24 and UM-UC-3 cells with 10 μg/mL polybrene (Sigma‒Aldrich, USA) at 37 °C overnight. Stable pools were selected with 800 μg/mL G418 for 7 days or 50 μg/mL hygromycin B for 5 days. The expression efficiency of the proteins was evaluated via Western blotting.

### Construction of organoids

The bladder cancer tissue was minced (1 mm^3^), and the primary cancer cells were collected via enzymatic digestion. The primary cancer cells were resuspended in Matrigel containing different amounts of type I collagen (low matrix stiffness: 0.25 mg/mL Col I; high matrix stiffness: 1.25 mg/mL Col I). Bladder cancer organoids were cultured in Bladder cancer organoid culture medium (Accuroid, Guangzhou, China) supplemented with 1% penicillin and streptomycin (Gibco) at 37 °C, and the medium was changed every 48 h.

### Measuring organoid formation

Representative bright-field images (20× magnification) were acquired every seven days. Fiji software was used to measure the diameter of the generated organoids and to plot growth curves.

### Single-cell RNA-seq data processing

The single-cell RNA sequencing data of human bladder cancer patients were downloaded from figshare with identifiers (https://figshare.com/articles/dataset/filtered_zip/23834670) [19]. Seurat (version 4.1.3) was used to perform the data processing. After quality control, normalization, cell clustering and cell type annotation, we performed the following analyses: compositional analysis, differential expression testing, gene set variation, and gene set enrichment.

### Atomic force microscopy (AFM)

Fresh tumor-adjacent, nonmuscle invasive bladder cancer (NIBC), and muscle invasive bladder cancer (IBC) tissues were collected and sliced after embedding with OCT compounds. The samples were centrifuged at 1500 rpm for 3 min to ensure cell stability and then soaked in PBS for atomic force microscopy. The elastic modulus of the bladder cancer tissue was measured via an atomic force microscope. Polystyrene microspheres with a radius of 2.5 μm were attached to a V-shaped, tipless cantilever (MLCT-O10) to assess the micromechanical properties of the extracellular matrix. Prior to the experiment, the elastic constant of the probe was calibrated via a thermal noise method. Force curves were obtained and analyzed via the built-in software Nanoscope Analysis 2.0.

### Niche collagen fiber assay

To detect the amount of collagen fibers in the cancer tissue, we performed Masson’s trichrome staining. Testicular tissue was fixed, embedded in paraffin, and cut into 10 μm sections. A Masson stain kit (Leagene, Beijing, China) was used according to the manufacturer’s instructions. Fiji software was used to measure the positive area of the cancer tissue to assess collagen deposition.

### Niche glycoprotein assay

To detect the amount of glycoproteins in the cancer tissue, the sections were stained via the periodic acid Schiff (PAS) staining method. A PAS stain kit (Leagene) was used according to the manufacturer’s instructions. Fiji software was used to measure the positive area of the cancer tissue to assess extracellular matrix deposition.

### Preparation of hydrogels with different matrix stiffnesses

Different ratios of acrylamide and polyacrylamide were used to construct two-dimensional matrix gels with varying stiffnesses, as shown in the literature [71]. After preparation, as described in the reference [72], a coagulant is added, and the resulting solution is quickly injected into a Western blot plate. After solidification, remove polyacrylamide gels from the plate and soak it in the PSB overnight. Next day, the appropriate size hydrogel was cut with a mold and placed into the culture plate. Expose the front and back sides of polyacrylamide gels to ultraviolet light for 1 h each time. Then, an appropriate amount of Sulfo-SANPAH (Macklin, Shanghai, China) was added to polyacrylamide gels, and it was exposed to ultraviolet light for 1 hour. Next, suction out Sulfo-SANPAH, wash twice with PBS, add rat tail collagen I (dissolved in 0.02 M acetic acid, 1:60), and incubate at 37 °C overnight. Morrow, place the culture plate back under the ultraviolet light for 1 h. Remove the solution and plant with cells.

### Immunofluorescence staining

The cells seeded on the hydrogel and bladder cancer organoids were fixed, permeabilized, blocked, and then incubated with primary antibodies overnight at 4 °C, followed by incubation with secondary antibodies in the dark for 1 h at room temperature. The primary and secondary antibodies used are described in Supplementary Table 1. Images were acquired via an LSM800 confocal microscope (Zeiss), DMI8 (Leica) or commercial SIM (HIS-SIM).

### Immunohistochemical staining

Sections of the paraffin-embedded tumor samples were incubated at 60 °C for 4 h in an oven, dewaxed with xylene and hydrated with an ethanol gradient (100–70%). After soaking in 3% H_2_O_2_ for 30 min, the slides were rinsed with PBS and incubated with the primary antibody overnight at 4 °C. The slides were subsequently rinsed and incubated with the corresponding secondary antibody for 30 min, followed by DAB and hematoxylin staining. The primary and secondary antibodies used can be found in Supplementary Table 1. The slides were then examined.

### Colony formation assay

The cells were seeded at a density of 1000 cells per 6-well plate (CORNING, NY, USA) in triplicate and cultured for 14 days. During colony growth, the culture medium was replaced every 3 days. Colonies with more than 50 cells were counted. The cells were counted under a light microscope.

### Cell growth assay

The cells were seeded on the hydrogels. Proliferation was measured via a CCK8 kit (Yeasen, Shanghai, China) according to the manufacturer’s instructions. A microplate reader was used to detect the absorbance at 450 nm, and the number of bacteria on each plate was counted.

### Clonal sphere formation assay

The cells were digested from hydrogels of different stiffnesses and resuspended in 12-well plates at a density of 5000 cells/mL for daily observation. Spheres were defined as free-floating spherical structures with a diameter >50 μm. Subsequently, primary spheres (day 10) were dissociated into single cells and replated under the same culture conditions as those used for the growth of primary spheres to generate secondary spheres. All spheres in the wells were counted, and their diameters were measured.

### Cell viability assay via calcein-AM/PI staining

The cells were seeded on hydrogels with different stiffnesses, and cell survival was detected 48 h later via a calcein/PI cell viability and cytotoxicity assay kit (Beyotime, Shanghai, China) according to the manufacturer’s instructions. Images of the cells were acquired immediately and analyzed via a DMI8 fluorescence microscope (Leica). The percentage of positive cells was determined via Fiji software.

### Matrigel invasion assay

For the Matrigel invasion assay, 3 × 10^5^ cells/well were seeded in the upper chamber, which was coated with Matrigel (CORNING). After 48 h at 37 °C and 5% CO_2_, the cells present on the lower surface of the insert were stained with crystal violet. The cells that invaded through the Matrigel-coated membrane were counted via microscopy.

### Luciferase reporter assay

Cells seeded into 96-well culture plates were transfected with the indicated pGL3-based plasmids for 24 h before reporter activity was assessed via a dual-luciferase reporter assay kit (Beyotime). Cotransfected Renilla luciferase measurements were used to normalize changes in transfection efficiency. Alternatively, TCF/LEF transcription activity was measured via TOP-FOP Flash reporters purchased from Beyotime (Shanghai, China). The cells were cotransfected with a pGL6-TA plasmid (10 ng/well) and either a TOP Flash plasmid or FOP Flash plasmid (30 ng/well) for 48 h before the luciferase activity was measured. The results are presented as the ratio of TOP flash activity to FOP flash activity.

### Transmission electron microscopy

The cell samples were fixed in 2.5% glutaraldehyde for 2 h at room temperature and then stored at 4 °C overnight. The samples were subsequently fixed with 1% osmium tetroxide for 1 h, washed, dehydrated through an ethanol gradient (30, 50, 70 and 95%, 5 min per step), embedded and polymerized at 60 °C for 48 h. Ultrathin 80-nm sections were cut and observed with a Tecnai 12 BioTwin transmission electron microscope (FEI Company, Eindhoven, The Netherlands) at 120 keV. For each biological experiment, 3 cells were selected respectively under low and high matrix stiffness conditions, and the sizes of their nuclear pores were measured. The average values were then calculated.

### Coimmunoprecipitation (Co-IP)

The cells were incubated on ice for 5 min with 300 μL of lysis buffer (Beyotime) (1 mM PMSF, protease inhibitor, and phosphatase inhibitor). The cells were scraped, the cellular debris was removed by centrifugation for 10 min at 12,000 rpm at 4 °C, and the protein concentration was determined. The cell supernatants were incubated with primary antibody overnight at 4 °C, followed by the addition of 50 μL of protein G agarose beads (Santa Cruz Biotechnology, CA, USA) for 2 h at 4 °C. The immunoprecipitates were washed two times with lysis buffer, separated by centrifugation for 2 min at 5000 rpm, and then heated with 5X sample buffer for electrophoresis and western blot analysis.

### Western blot

The cell suspensions were collected and washed three times with PBS. Then, the cells were lysed in 1X RIPA buffer with 1X protein inhibitor solution for at least 30 min and centrifuged at 12,000 × *g* for 10 min at 4 °C to remove cell debris. The protein mixture was aspirated, and the protein mixture was mixed with 4X loading buffer (Bio-Rad). After the total protein concentration was measured via a BCA protein assay kit (Beyotime), equal amounts of total protein were resolved via SDS‒PAGE (Epizyme, Shanghai, China) and then electrotransferred to a 0.45 μm pore size polyvinylidene difluoride membrane (Millipore, MA, USA). After blocking with 5% BSA, the membrane was incubated with primary antibodies at 4 °C overnight, followed by incubation with HRP-conjugated secondary antibodies for 1 h at room temperature. The primary and secondary antibodies used can be found in Supplementary Table 1. A chemiluminescent substrate (Millipore) was used to detect the signal intensity. Bands from at least three independent blots were quantified via Fiji software.

### RNA isolation and real-time quantitative PCR

Total RNA was extracted from testis tissue or cells via TRIzol reagent (Thermo Fisher Scientific, MA, USA) according to the manufacturer’s protocol. Quantification was performed with a NanoDrop 8000 spectrophotometer, and 1 μg of total RNA was reverse transcribed with a HiFiScript All-in-one RT Master Mix Kit (Cwbiotech, Beijing, China). cDNAs were used as the template for real-time quantitative PCR (qPCR) reactions with SuperStar Universal SYBR Master Mix (Cwbiotech). All samples were run in triplicate, and the results were normalized to the relative mRNA levels of GAPDH. The primers designed and used for qPCR are described in Supplementary Table 2.

### Xenograft study

Athymic nu/nu female mice (n = 5, each group) were injected subcutaneously in the dorsal flank with T24 cells (3 × 10^6^ cells/mouse) in 100 µl of PBS or Matrigel. IWR-1 (MedChemExpress, NJ, USA) or cilengitide (MedChemExpress) was injected into the surrounding area of the tumor (every 3 days) on the seventh day. The tumor volume was measured via a standard caliper (reaching a tumor volume of 1500 mm^3^ as the endpoint of comparison) and was calculated via the following formula: V = L×W^2^/2. All the mice were sacrificed via euthanasia after 25 days, and the xenograft tumors were collected for immunohistochemistry, qPCR and Western blotting.

### Statistical analysis

All experiments were carried out with at least three biological replicates, and successful reproducibility is shown. All the data are reported as the means ± SEMs of at least three independent experiments. The sample sizes are all presented in the figure legends. Statistical analysis between two groups was performed via an unpaired t test. Statistical analysis between multiple groups was performed via one-way ANOVA. All the data were analyzed via GraphPad Software. A two-sided p value < 0.05 was considered statistically significant. The level of significance was defined as p < 0.05 (*), p < 0.01 (**), and p < 0.001 (***).

## Supplementary information

Supplementary Figure 1

Supplementary Figure 2

Supplementary Figure 3

Supplementary Figure 4

Supplementary Figure 5

Supplementary Figure 6

Supplementary Figure 7

Supplementary Figure 8

Supplementary Figure 9

Supplementary Figure 10

Supplementary Figure legends

Supplementary Table

Uncropped western blots

## Data Availability

The datasets generated and/or analysed during the current study are available in the Figshare, https://figshare.com/articles/dataset/filtered_zip/23834670.

## Code availability

No new algorithms were developed for this article. All code generated for analysis is available from the authors upon request.
