# Supplementary material for: Matrix stiffness maintains bladder cancer stemness via integrin-nuclear skeleton axis
Source: Cell Death Dis. 2025 Dec 12;16(1):887. doi: 10.1038/s41419-025-08222-7 (PMC12700999; doi:10.1038/s41419-025-08222-7)
Supplement: Supplementary file 11 — Supplementary Figure legends [file 41419_2025_8222_MOESM11_ESM.docx]

**Supplemental Information**

**Matrix stiffness maintains bladder cancer stemness via integrin-nuclear skeleton axis**

**Tao et al.**

**Supplementary Fig. 1 | High matrix stiffness is associated with bladder cancer stemness and poor prognosis.**

(A) The heatmap of the normalized mean expression of key signature genes for each major cell type is shown in **Fig. 1A**.

(B) The expression levels of genes involved in extracellular matrix secretion.

(C) The histogram shows that the DEGs of bladder cancer cells in the low-grade group and high-grade group were significantly enriched in the gene sets.

(D) The overall survival of bladder cancer patients with COL1A2, LOX and PLOD2 expression was calculated via Kaplan–Meier analysis, and COL1A2, LOX and PLOD2 expression was significantly correlated with overall survival (p < 0.05, Kaplan–Meier).

**Supplementary Fig. 2 | The stemness of cancer cells increases with the progression of bladder cancer.**

(A) Representative image of IHC of human BC samples stained for CD44, OCT4, SOX2 and NANOG. Magnification ×10 (lower, bar = 100 μm) and ×40 (upper, bar = 10 μm). (n = 5 biological replicates for each group; unpaired t test).

(B) The histogram indicates the percentage of CD44-, OCT4-, SOX2- and NANOG-positive tissues in Adjacent, NIBC and IBC samples.

Data are represented as mean ± SEM. In all bar graphs, each dot represents one biological replicate. *: p < 0.05, **: p < 0.01, ***: p < 0.001, ns, no significance by one-way ANOVA (B).

**Supplementary Fig. 3 | High matrix stiffness promotes the stemness of bladder cancer cells.**

(A**–**B) Western blot analysis and quantification of stemness marker expression in UM-UC-3 cells cultured on 5 kPa, 16 kPa and 30 kPa polyacrylamide gels.

(C**–**D) A CCK8 assay was used to detect and analyze the IC50 of cisplatin (DDP) in T24 (C) and UM-UC-3 (D) cells.

(E**–**F) T24 and UM-UC-3 cells were treated with DDP at the indicated concentrations for 24 h. Then, the cells were counted.

Data are represented as mean ± SEM. In all bar graphs, each dot represents one biological replicate. *: p < 0.05, **: p < 0.01, ***: p < 0.001, ns, no significance by unpaired Student’s t test (E and F) or one-way ANOVA (B).

**Supplementary Fig. 4 | Inhibition of the Wnt pathway effectively reduces bladder cancer cell stemness.**

(A) qPCR analysis of the relative mRNA expression of Hedgehog, Wnt and Notch pathway downstream genes in UM-CU-3 cells cultured on 5 kPa and 30 kPa polyacrylamide gels.

(B) Western blot analysis of stemness marker expression in UM-UC-3 and UM-UC-3 cells treated with IWR-1 cultured on 5 kPa and 30 kPa polyacrylamide gels.

(C) qPCR analysis of the relative mRNA expression of stemness markers in UM-CU-3 and UM-UC-3 cells treated with IWR-1 cultured on 5 kPa and 30 kPa polyacrylamide gels.

(D) Transwell Matrigel invasion assay of representative images of the indicated T24 and UM-UC-3 cells treated with IWR-1 cultured on 5 kPa and 30 kPa polyacrylamide gels. (bar = 200 μm).

(E) Quantitative analyses of cell invasion through Matrigel-coated membranes.

(F) Spheres of representative images of the indicated T24 and UM-UC-3 cells treated with IWR-1 cultured on 5 kPa and 30 kPa polyacrylamide gels. (bar = 100 μm).

(G**–**H) Histograms showing the mean numbers and diameters of spheres cultured.

(I) UM-UC-3 and UM-UC-3 cells treated with IWR-1 were treated with DDP at the indicated concentrations for 24 h. Then, the cell numbers were counted.

Data are represented as mean ± SEM. In all bar graphs, each dot represents one biological replicate. *: p < 0.05, **: p < 0.01, ***: p < 0.001, ns, no significance by one-way ANOVA (E and G-I).

**Supplementary Fig. 5 | Interfering with the expression of β-catenin effectively reduces bladder cancer cell stemness.**

(A) qPCR analysis of the relative mRNA expression of stemness markers in T24 and T24 cells treated with Si β-catenin cultured on 5 kPa and 30 kPa polyacrylamide gels. (B) Spheres of representative images of the indicated T24 and UM-UC-3 cells treated with Si β-catenin cultured on 5 kPa and 30 kPa polyacrylamide gels. (bar = 100 μm). (C) Histograms showing the mean numbers and diameters of spheres cultured.

(D) Colony formation assay of representative images of the indicated T24 and UM-UC-3 cells treated with Si β-catenin cultured on 5 kPa and 30 kPa polyacrylamide gels.

(E) Quantitative analyses of the number of cell colonies.

(F) T24 and T24 cells treated with Si β-catenin were treated with DDP at the indicated concentrations for 24 h. Then, the cell numbers were counted.

Data are represented as mean ± SEM. In all bar graphs, each dot represents one biological replicate. *: p < 0.05, **: p < 0.01, ***: p < 0.001, ns, no significance by one-way ANOVA (A, C and E-F).

**Supplementary Fig. 6 | Promotion of β-catenin translocation into the nucleus can increase bladder cancer cell stemness under conditions of low matrix stiffness.**

(A) qPCR analysis of the relative mRNA expression of Wnt pathway-related genes in UM-UC-3 cells cultured on 5 kPa and 30 kPa polyacrylamide gels.

(B) Representative transmission electron microscopy (TEM) images of UM-UC-3 cells cultured on 5 kPa and 30 kPa polyacrylamide gels. (left: bar = 500 nm, right: bar = 200 nm).

(C) Quantitative analysis of the nuclear pore size in **(B)**.

(D**–**E) Western blot analysis and quantification of β-catenin expression in the nucleus and cytoplasm of UM-UC-3 cells treated with CHD and Pitstop2 cultured on 5 kPa and 30 kPa polyacrylamide gels.

(F) qPCR analysis of the relative mRNA expression of stemness markers in UM-UC-3 cells treated with CHD and Pitstop2 cultured on 5 kPa polyacrylamide gels.

(G) Representative Calcein-AM/PI staining images of the indicated UM-UC-3 cells treated with CHD and Pitstop2 cultured on 5 kPa polyacrylamide gels. (survival, green+; dead, red+). (bar = 100 μm).

(H) Quantitative statistical graph of **(G)**.

(I) Spheres of representative images of the indicated UM-UC-3 cells treated with CHD and Pitstop2 cultured on 5 kPa polyacrylamide gels. (bar = 100 μm).

(J) Histograms showing the mean diameters of the cultured spheres.

Data are represented as mean ± SEM. In all bar graphs, each dot represents one biological replicate. *: p < 0.05, **: p < 0.01, ***: p < 0.001, ns, no significance by unpaired Student’s t test (A and C) or one-way ANOVA (E, F, H and J).

**Supplementary Fig. 7 | Integrin and LINC complex mediate the transmission of biomechanical signals within bladder cancer cells.**

(A) qPCR analysis of the relative mRNA expression of integrin in T24 cells cultured on 5 kPa and 30 kPa polyacrylamide gels.

(B-C) Western blot analysis and quantification of integrin and Rho/ROCK pathway expression in T24 cells cultured on 5 kPa and 30 kPa polyacrylamide gels.

(D-E) Western blot analysis of FAK/Src pathway and active β-catenin expression in T24 and T24 cells treated with cilengitide (C) cultured on 5 kPa and 30 kPa polyacrylamide gels.

(F) qPCR analysis of the relative mRNA expression of LINC complex in T24 cells cultured on 5 kPa and 30 kPa polyacrylamide gels.

1. H) Western blot analysis and quantification of β-catenin expression in the nucleus and cytoplasm of T24 cells treated with Si Nesprin2 and cilengitide cultured on 5 kPa and 30 kPa polyacrylamide gels.

(I) qPCR analysis of the relative mRNA expression of stemness markers in T24 cells treated with Si β-catenin and cilengitide cultured on 5 kPa and 30 kPa polyacrylamide gels.

Data are represented as mean ± SEM. In all bar graphs, each dot represents one biological replicate. *: p < 0.05, **: p < 0.01, ***: p < 0.001, ns, no significance by unpaired Student’s t test (A, C and F) or one-way ANOVA (E and H-I).

**Supplementary Fig. 8 | Interference with Lamin A/C expression can reduce bladder cancer cell stemness under high matrix stiffness.**

(A**–**B) Western blot analysis and quantification of lamin expression in UM-UC-3 cells cultured on 5 kPa and 30 kPa polyacrylamide gels.

(C) UM-UC-3 cells were cultured on 5 kPa and 30 kPa polyacrylamide gels. The lysates were incubated with an anti-Lamin A/C antibody for 12 h and then conjugated with agarose. Bound proteins were analyzed by immunoblotting with an anti-β-catenin antibody. Input samples were taken prior to immunoprecipitation and immunoblotted with the indicated antibodies.

(D) Quantitative statistical graph of **(C)**.

(E**–**F) Co-IP assay analysis and quantification of β-catenin expression in UM-UC-3 cells with or without Lamin A/C depletion cultured on 30 kPa polyacrylamide gels. (G**–**H) Western blot analysis and quantification of β-catenin expression in the nucleus and cytoplasm of UM-UC-3 cells with or without Lamin A/C depletion and treated with HLY78 cells cultured on 5 kPa and 30 kPa polyacrylamide gels.

(I) qPCR analysis of the relative mRNA expression of genes downstream of the Wnt pathway in UM-UC-3 cells with or without Lamin A/C depletion cultured on 30 kPa polyacrylamide gels.

(J**–**K) Representative Calcein-AM/PI staining images of the indicated T24 and UM-UC-3 cells with or without Lamin A/C depletion cultured on 30 kPa polyacrylamide gels. (survival, green+; dead, red+). (bar = 100 μm). **(J)** Quantitative statistical graph.

Data are represented as mean ± SEM. In all bar graphs, each dot represents one biological replicate. *: p < 0.05, **: p < 0.01, ***: p < 0.001, ns, no significance by unpaired Student’s t test (B and D) or one-way ANOVA (F and H-J).

**Supplementary Fig. 9 | Inhibiting the Wnt pathway and mechanotransduction effectively inhibits the stemness of xenograft tumors.**

(A**–**B) Western blot analysis and quantification of stemness marker expression in xenograft tumors from *in vivo* Model I.

(C) qPCR analysis of the relative mRNA expression of genes downstream of the Wnt pathway in xenograft tumors from *in vivo* Model I.

(D**–**E) Western blot analysis and quantification of stemness marker expression in xenograft tumors from *in vivo* Model II.

(F) qPCR analysis of the relative mRNA expression of genes downstream of the Wnt pathway in xenograft tumors from *in vivo* Model II.

Data are represented as mean ± SEM. In all bar graphs, each dot represents one biological replicate. *: p < 0.05, **: p < 0.01, ***: p < 0.001, ns, no significance by one-way ANOVA (B, C and E-F).

**Supplementary Fig. 10 | High matrix stiffness promotes bladder cancer organoid regrowth.**

(A) Thermal map of matrix stiffness at the T2 and T3 stages of bladder cancer tissue. (scale bar = 700 μm).

(B) Representative immunofluorescence images of Ki67 in organoids cultured with or without IWR-1 for three passages in organoid Model I (bar = 25 μm).

(C) qPCR analysis of the relative mRNA expression of genes downstream of the Wnt pathway in Model I organoids.

(D) qPCR analysis of the relative mRNA expression of stemness markers in Model II organoids.

(E) qPCR analysis of the relative mRNA expression of genes downstream of the Wnt pathway in Model II organoids.

(F) Representative immunofluorescence images of Ki67 in organoids cultured with or without Cilengitide (C) for three passages in Model II. (bar = 25 μm).

Data are represented as mean ± SEM. In all bar graphs, each dot represents one biological replicate. *: p < 0.05, **: p < 0.01, ***: p < 0.001, ns, no significance by one-way ANOVA (C-E).
