# Supplementary material for: Matrix stiffness maintains bladder cancer stemness via integrin-nuclear skeleton axis
Source: Cell Death Dis. 2025 Dec 12;16(1):887. doi: 10.1038/s41419-025-08222-7 (PMC12700999; doi:10.1038/s41419-025-08222-7)
Supplement: Supplementary file 12 — Supplementary Table [file 41419_2025_8222_MOESM12_ESM.docx]

**Supplemental Information**

**Matrix stiffness maintains bladder cancer stemness via integrin-nuclear skeleton axis**

**Tao et al.**

**Supplementary Table 1 | Antibodies, Chemical reagents and Kits**

| **Antibodies** | **Company** | **Cat. No** |
| --- | --- | --- |
| CD44 | proteintech | 15675-1-AP |
| OCT4 | proteintech | 11263-1-AP |
| NANOG | proteintech | 14295-1-AP |
| SOX2 | proteintech | 11064-1-AP |
| c-Myc | CST | 5605T |
| c-Jun | CST | 9165T |
| Cyclin D1 | CST | 55506T |
| p-LRP6 | CST | 2568T |
| p-GSK3β | CST | 5558T |
| β-Catenin | CST | 8480S |
| Active β-Catenin | CST | 8814S |
| Integrin α2 | proteintech | 30703-1-AP |
| Integrin β3 | proteintech | 18309-1-AP |
| F-actin | Invitrogen | MA1-80729 |
| LIMK1 | CST | 3842S |
| p-LIMK1 | Invitrogen | PA5-37629 |
| p-FAK | CST | 8556T |
| FAK | CST | 3285T |
| p-Src | CST | 6943T |
| Src | CST | 2109T |
| LEF1 | Santa Cruz | sc-374412 |
| Lamin B1 | Santa Cruz | sc-374015 |
| H3 | CST | 14269S |
| Lamin A/C | Santa Cruz | sc-376248 |
| p-Lamin A/C | CST | 13448T |
| IgG | proteintech | B900620 |
| Ki67 | Abcam | ab16667 |
| GAPDH | proteintech | 60004-1-Ig |
| Goat Anti-Rabbit IgG H&L (HRP) | Abcam | ab205718 |
| Goat Anti-Mouse IgG H&L (HRP) | Abcam | ab205719 |
| Goat anti-mouse IgG conjugated to Alexa Fluor™ 488 | Invitrogen | A-11001 |
| Goat anti-mouse IgG conjugated to Alexa Fluor™ 555 | Invitrogen | A-21422 |
| Goat anti-rabbit IgG conjugated to Alexa Fluor™ 488 | Invitrogen | A-11008 |
| Goat anti-rabbit IgG conjugated to Alexa Fluor™ 555 | Invitrogen | A-21428 |
| **Chemical reagents** |  |  |
| IWR-1 | MedChemExpress | HY-12238 |
| trans-1,2-Cyclohexanediol | Sigma | 141712 |
| Pitstop2 | Abcam | ab120687 |
| HLY78 | MedChemExpress | HY-122816 |
| Cilengitide | MedChemExpress | HY-16141 |
| Acryiamide | Acryiamide | A100341-0500 |
| Bis-acrylamide | Shanghai yuanye Bio-Technology | S14002 |
| Ammonium persulfate (APS) | Sangon Biotech | A100486-0100 |
| HEPES | Cytiva | SH30237.01 |
| TEMED | Phygene | PH0341 |
| Collagen, Type I , from rat tail | Yeasen | 40125ES50 |
| SANPAH | Macklin | 102568-43-4 |
| Matrigel | CORNING | 356237 |
| Gel digestion enzymes | Accurate Biotechnology | GXDLFA |
| DAPI Solution | Sigma | D9542-1 mg |
| TRIzol | Thermo | 15596018 |
| PMSF Solution (100 mM) | Beyotime | ST507-10 ml |
| TOPFlash | Beyotime | D2501 |
| FOPFlash | Beyotime | D2503 |
| Lentivirus | RiboBio | N/A |
| protein G agarose beads | Santa Cruz | sc-2003 |
| **Kits** |  |  |
| Masson Stain Kit | Leagene | DC0033 |
| PAS Stain Kit | Leagene | DG0005 |
| Cell Counting Kit (CCK-8) | Yeasen | 40203ES80 |
| Calcein/PI Cell Viability/Cytotoxicity Assay Kit | Beyotime | C2015S |
| BCA Protein Assay Kit | Beyotime | P0012S |
| riboFECT CP Transfection Kit | RiboBio | C10511-05 |
| HiFiScript All-in-one RT Master Mix Kit | Cwbiotech | CW3371 |

**Supplementary Table 2 | Primers used for real-time PCR**

| hCD44-Forward | GAATCCCTGCTACCAATAGGAAT |
| --- | --- |
| hCD44-Reverse | GGTCCTGCTTTCCTTCGTGT |
| hNANOG-Forward | AATACCTCAGCCTCCAGCAGATG |
| hNANOG-Reverse | TGCGTCACACCATTGCTATTCTTC |
| hSOX2-Forward | AAATGGGAGGGGTGCAAAAGAGGAG |
| hSOX2-Reverse | CAGCTGTCATTTGCTGTGGGTGATG |
| hPOU5F1-Forward | CCCGAAAGAGAAAGCGAACCA |
| hPOU5F1-Reverse | CAGAACCACACTCGGACCAC |
| hHHIP-Forward | AATGCAGAGCCACGGTACAA |
| hHHIP-Reverse | GCTGGCTCACATTTTGCAGT |
| hHHAT-Forward | GAAGTGCCGAAAGAGGGGTG |
| hHHAT-Reverse | GCTGAGAGTTTCTCCGGGC |
| hMYCN-Forward | ATCCTGGCTAGAGGAGACCC |
| hMYCN-Reverse | CCGACCTCCAACACGGC |
| hEGF-Forward | CTTGGGAGCCTGAGCAGAAA |
| hEGF-Reverse | GCACAAGTGTGACTGGAGGT |
| hGLI3-Forward | GACATCATGGAGGCCCAGTC |
| hGLI3-Reverse | TGTCCAGGACTTTCATCCTCATT |
| hCCND1-Forward | GAGGAGCTGCTGCAAATGG |
| hCCND1-Reverse | CAATGAAATCGTGCGGGGTC |
| hMYC-Forward | GGAAAACCAGCAGCCTCC |
| hMYC-Reverse | ACCGAGTCGTAGTCGAGGTC |
| hJUN-Forward | ATCAAGGCGGAGAGGAAGCG |
| hJUN-Reverse | TGAGCATGTTGGCCGTGGAC |
| hMMP7-Forward | AGTGGTCACCTACAGGATCG |
| hMMP7-Reverse | GGGATCTCTTTGCCCCACAT |
| hTCF7-Forward | CCAAGAATCCACCACAGGAGG |
| hTCF7-Reverse | GCAGCCTAGAGCACTGTCAT |
| hTP53-Forward | AAAGTCTAGAGCCACCGTCC |
| hTP53-Reverse | GCAGTCTGGCTGCCAATCC |
| hVIM-Forward | CCTCCGGGAGAAATTGCAGG |
| hVIM-Reverse | GCGTTCAAGGTCAAGACGTG |
| hHEY1-Forward | GCTTTTGAGAAGCAGGGATCT |
| hHEY1-Reverse | TCCTGCCGTATGCAGCATTT |
| hHES1-Forward | ACGACACCGGATAAACCAAAGA |
| hHES1-Reverse | ATGCCGCGAGCTATCTTTCT |
| hCDKN1A-Forward | AGCGATGGAACTTCGACTTTG |
| hCDKN1A-Reverse | CGAAGTCACCCTCCAGTGGT |
| hHES6-Forward | GTGAGGATGAGGACGGCTG |
| hHES6-Reverse | CTTGGCCTGCACCTCGG |
| hCCND3-Forward | GAGGTGCAATCCTCTCCTCG |
| hCCND3-Reverse | GCTGCTCCTCACATACCTCC |
| hGAPDH-Forward | TCGGAGTCAACGGATTTGGT |
| hGAPDH-Reverse | TGAAGGGGTCATTGATGGCA |
| hWNT1-Forward | AGATCGTCAACCGAGGCTGT |
| hWNT1-Reverse | ACACGTGCAGGATTCGATGG |
| hLRP6-Forward | TGCATGGACTGAGGAACGTC |
| hLRP6-Reverse | CAAAACAAAGGGCTGGGTCC |
| hGSK3B-Forward | CCTCAGGAGTGCGGGTCTTC |
| hGSK3B-Reverse | GTTAGTCGGGCAGTTGGTGT |
| hAXIN2-Forward | GCCCTGCTGTAAAAGAGAGGA |
| hAXIN2-Reverse | GCTCTTCCCACTGAGTCTGG |
| hCTNNB1-Forward | GGTCGAGGACGGTCGGA |
| hCTNNB1-Reverse | CCAACTCCATCAAATCAGCTTG |
| hLEF1-Forward | GCCTACATCTGAAACATGGTGG |
| hLEF1-Reverse | TGTTCCTTTGGGGTCGACTG |
| hTCF4-Forward | GTTGCCACCAGCAGAGTC |
| hTCF4-Reverse | AGGCCTCCTTCGGGGATTAT |
| hITGA2-Forward | ATTCTCCCTGCCGGTTGATG |
| hITGA2-Reverse | CAGGGTAGCCTACATCGCAG |
| hITGA6-Forward | TGTGCTTGCTCTACCTGTCG |
| hITGA6-Reverse | ACGAGCAACAGCCGCTT |
| hITGB3-Forward | AGATTGGAGACACGGTGAGC |
| hITGB3-Reverse | GCCCACGGGCTTTATGGTAA |
| hITGB4-Forward | CTGCAGCCCCATCTCCTAGC |
| hITGB4-Reverse | GCCATCCTCTTCCTCCCTCT |
| hSUN1-Forward | ACGTATGCGCTCAGTTCCAG |
| hSUN1-Reverse | CGGGACATCCGTGGAGAATC |
| hSUN2-Forward | CGCGGCAGCAGATTCTCTT |
| hSUN2-Reverse | TCATCGTCACCCTGGGAGTA |
| hSYNE1-Forward | GGTGGTCCCGGTATAAAGGC |
| hSYNE1-Reverse | CCCAGCCGCCCTCCT |
| hSYNE2-Forward | CTTGCAGGGAACCCAGAACC |
| hSYNE2-Reverse | TGCTCTGAACTGCTTTGCTC |
| hSYNE3-Forward | GGATGGAAGTGGATTCGGGG |
| hSYNE3-Reverse | TGTAGAAGATTTGCGCGTCG |
