# Supplementary material for: Matrix stiffness maintains bladder cancer stemness via integrin-nuclear skeleton axis
Source: Cell Death Dis. 2025 Dec 12;16(1):887. doi: 10.1038/s41419-025-08222-7 (PMC12700999; doi:10.1038/s41419-025-08222-7)

Figure 2A

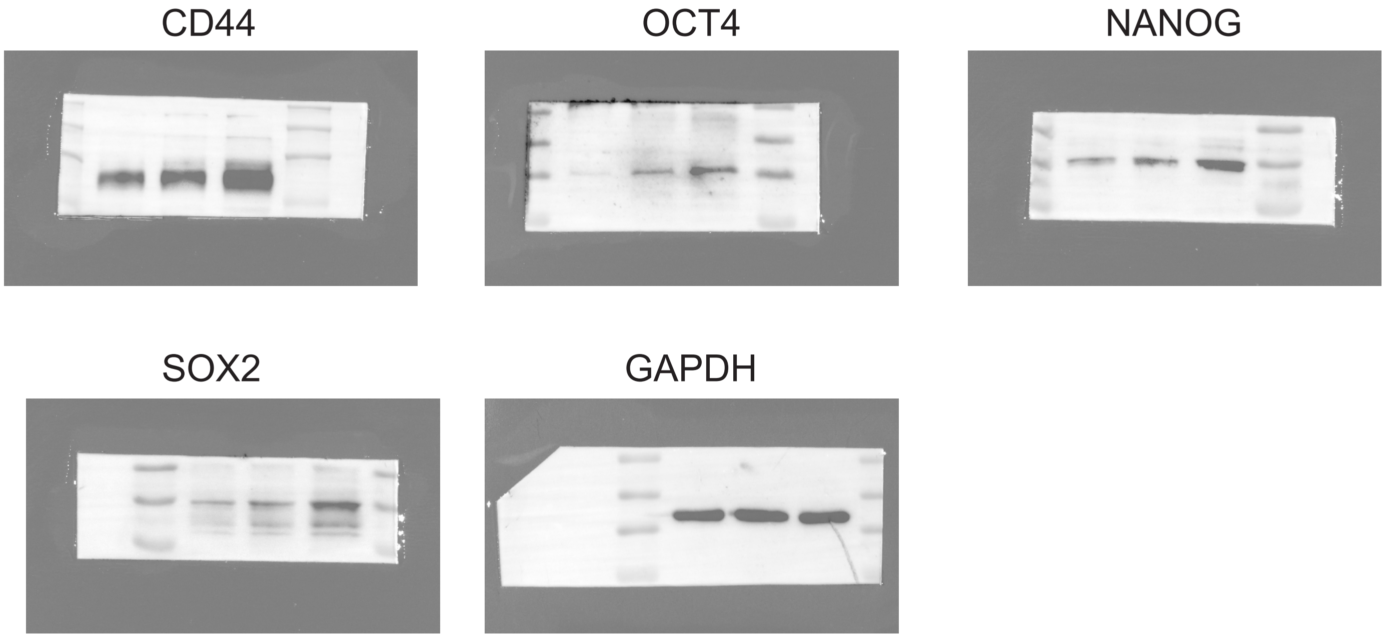

Figure 3B

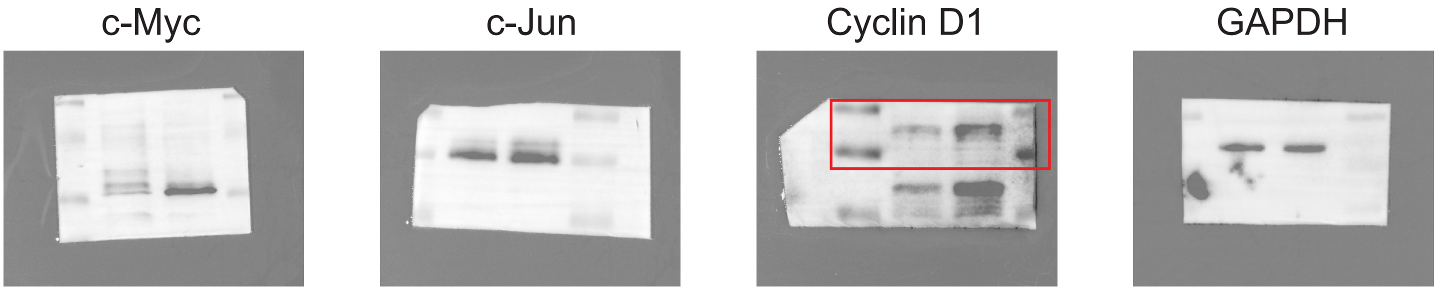

Figure 4B

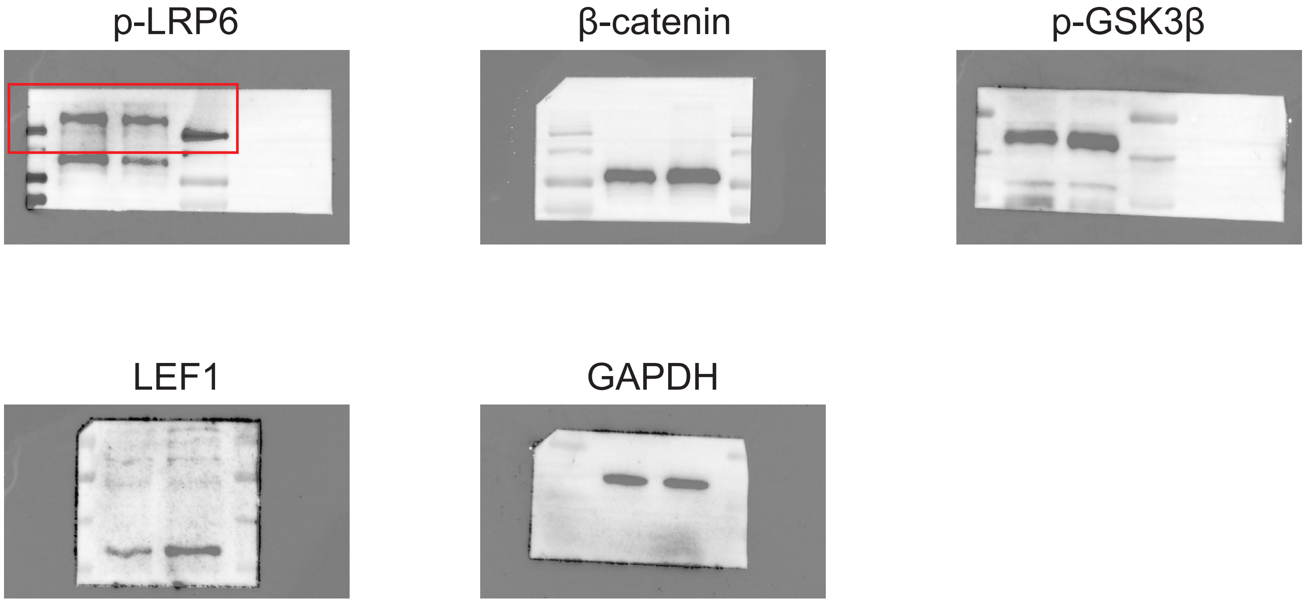

Figure 4J

Cytosolic β-catenin

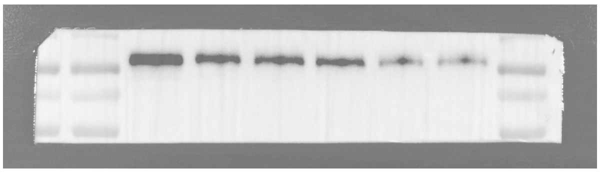

Nuclear β-catenin

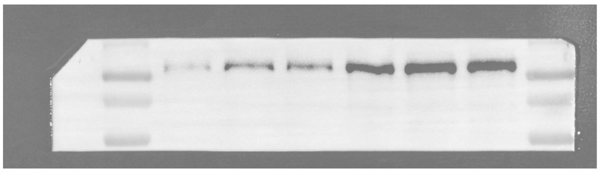

GAPDH

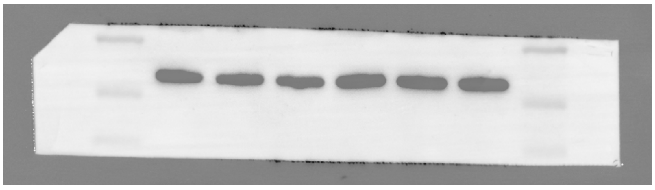

H3

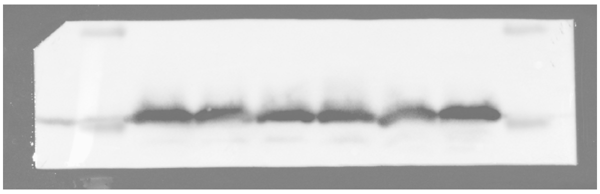

Figure 5A

Lamin A/C

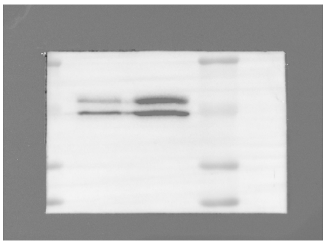

p-Lamin A/C

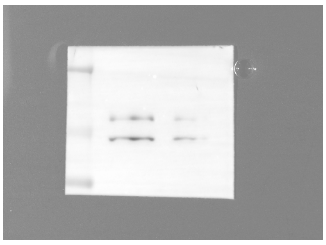

Lamin B1

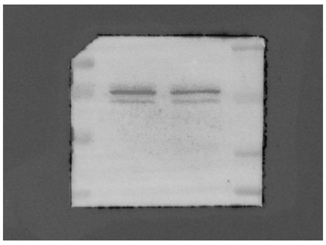

GAPDH

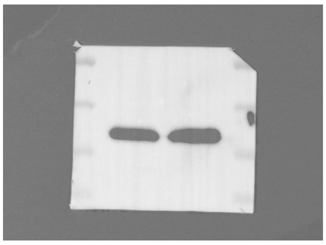

Figure 5C

IB β-catenin

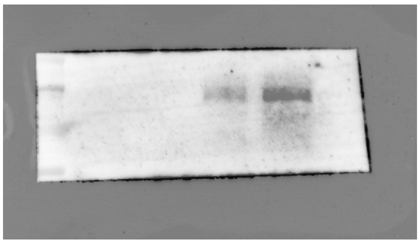

IB Lamin A/C

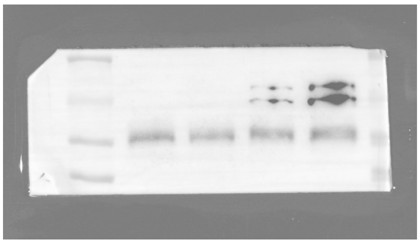

Input β-catenin

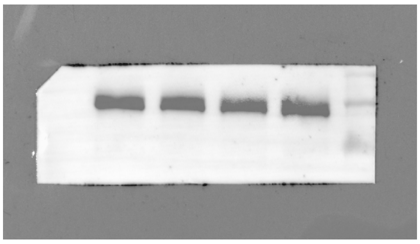

Input Lamin A/C

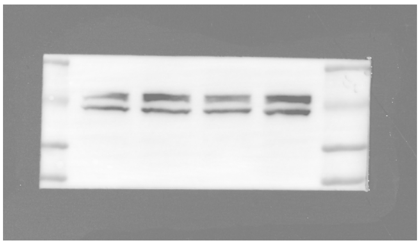

GAPDH

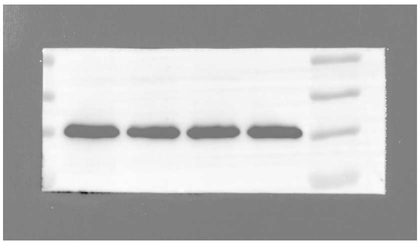

Figure 5H

IB β-catenin

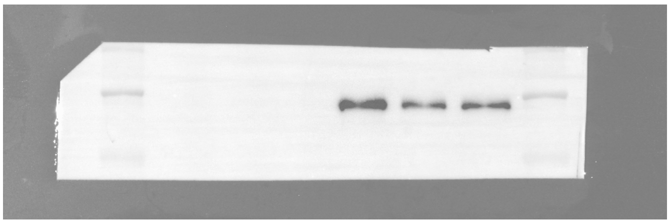

IB Lamin A/C

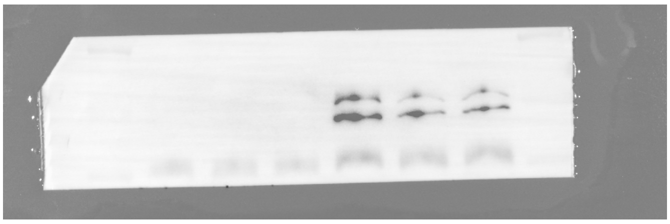

Input β-catenin

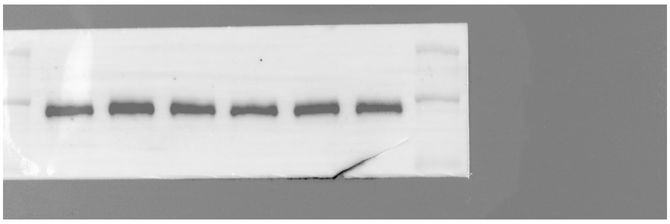

Input Lamin A/C

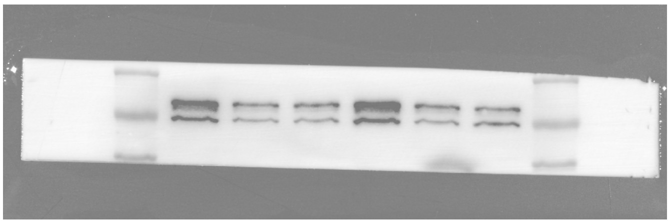

GAPDH

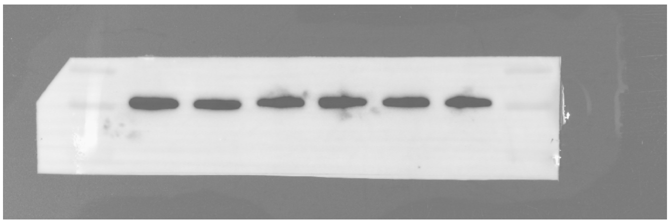

Figure 5J

LEF1

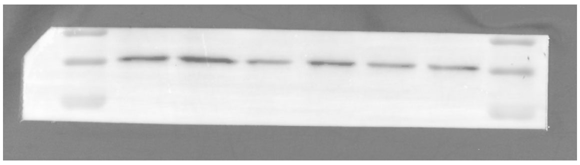

Lamin A/C

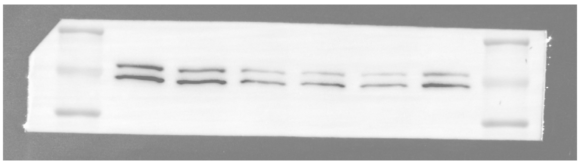

GAPDH

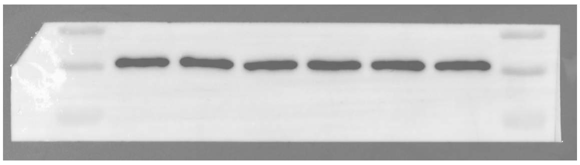

Figure 5M

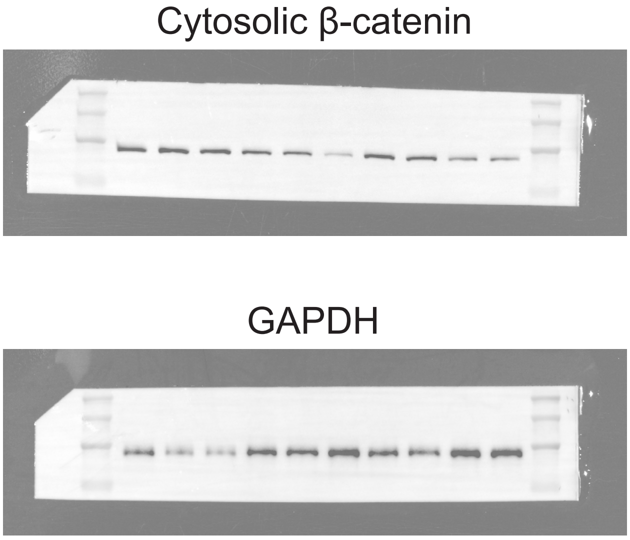

Figure 7C

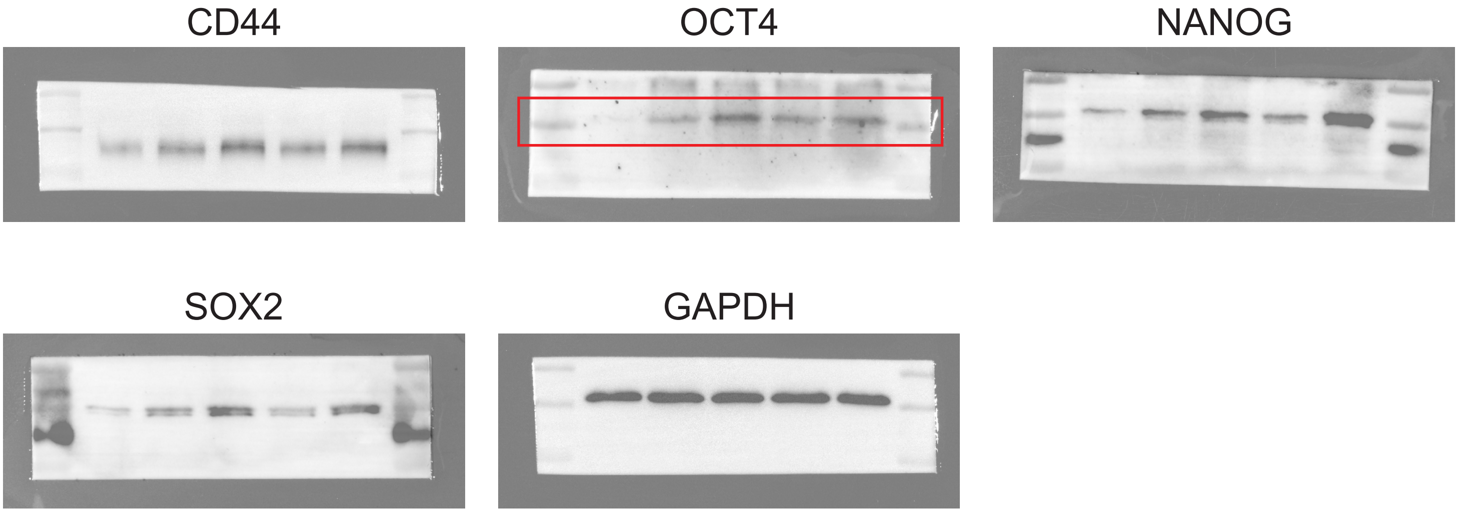

Figure 7H

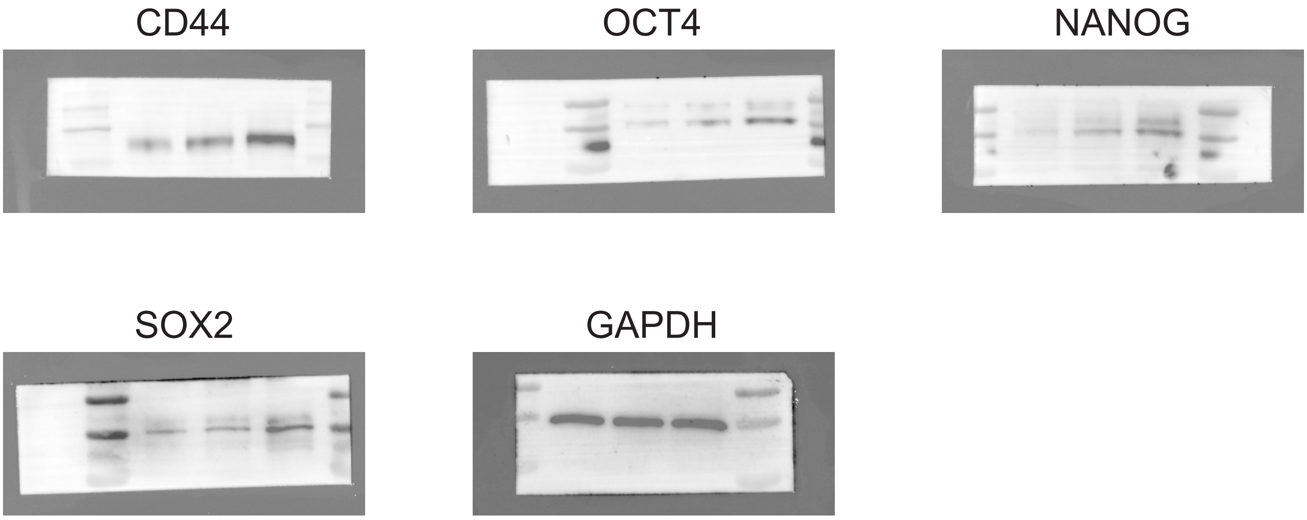

Supplementary Figure 3A

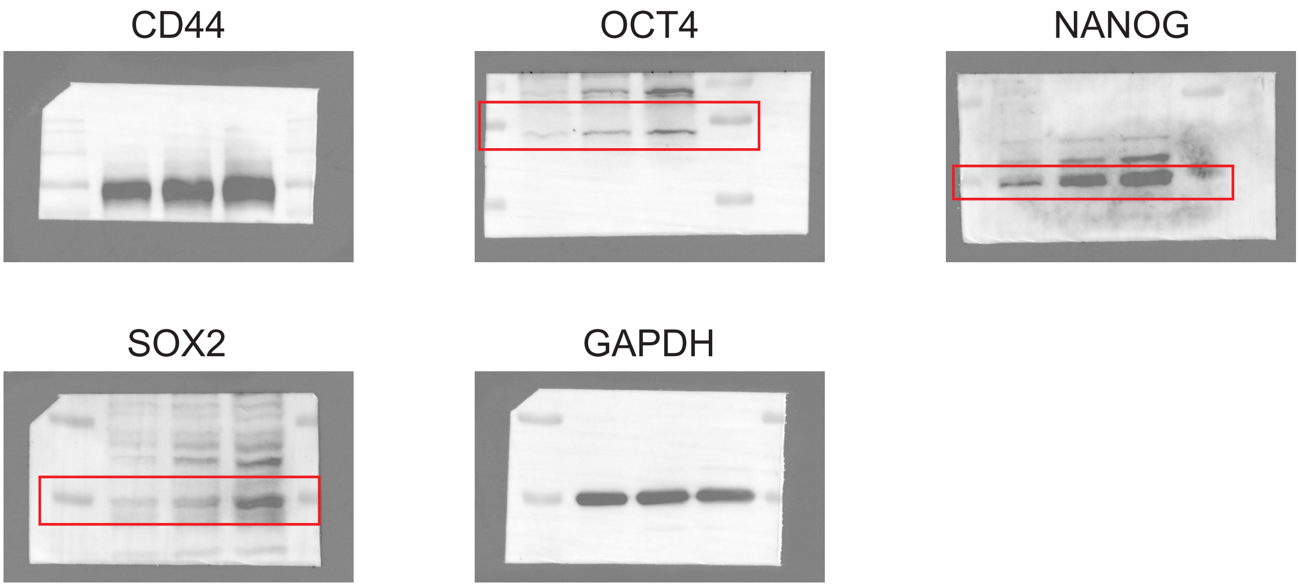

Supplementary Figure 4B

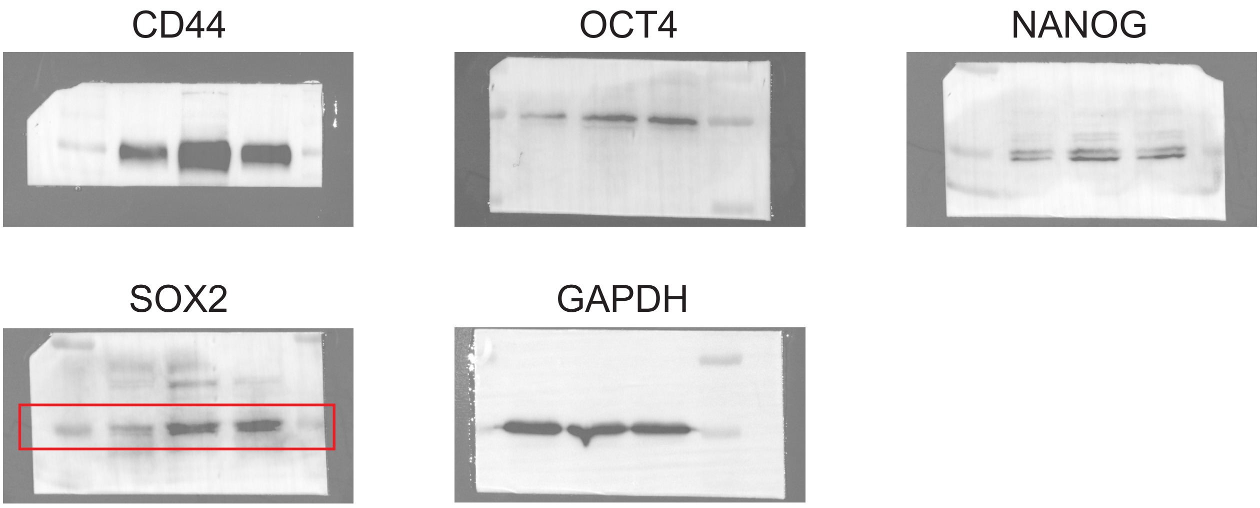

Supplementary Figure 6D

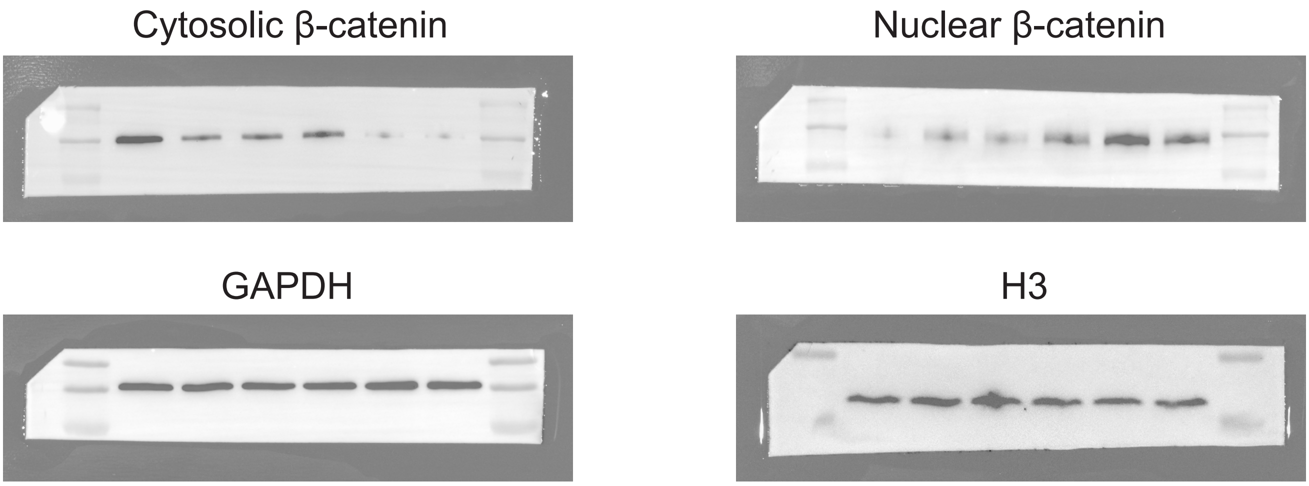

Supplementary Figure 7B

Integrin α2

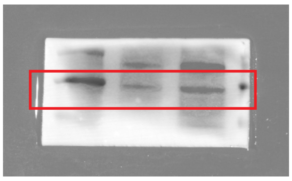

Integrin β3

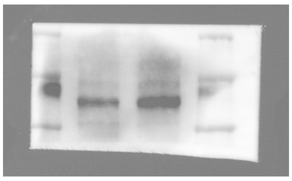

F-actin

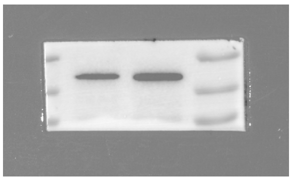

p-LIMK1

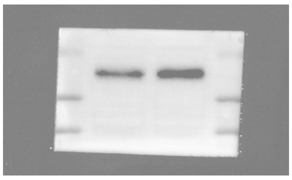

LIMK1

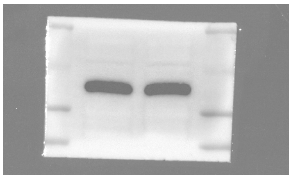

GAPDH

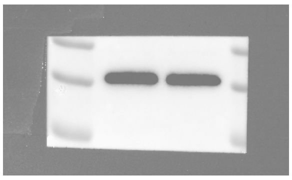

Supplementary Figure 7D

p-FAK

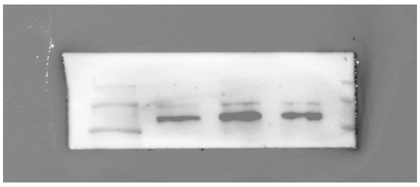

FAK

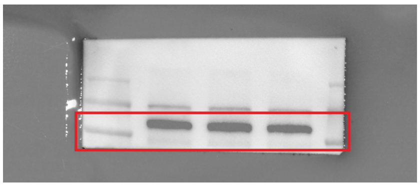

p-Src

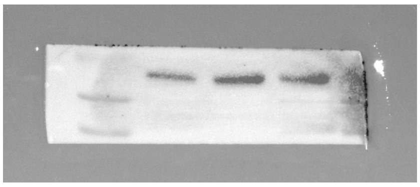

Src

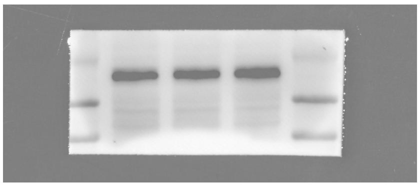

Active β-catenin

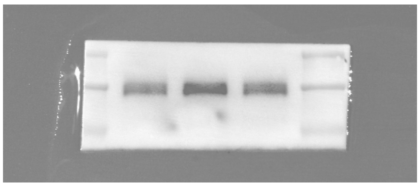

GAPDH

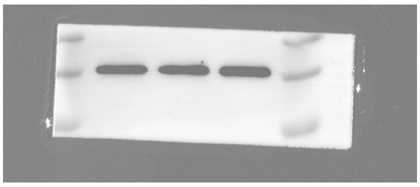

Supplementary Figure 7G

Cytosolic β-catenin

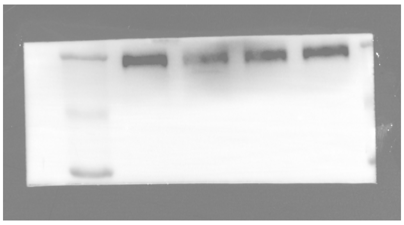

Nuclear β-catenin

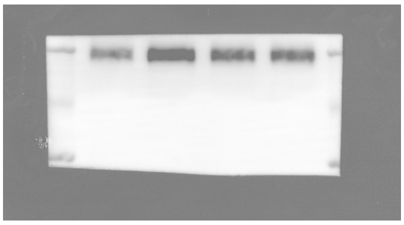

GAPDH

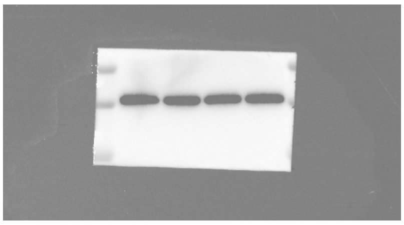

H3

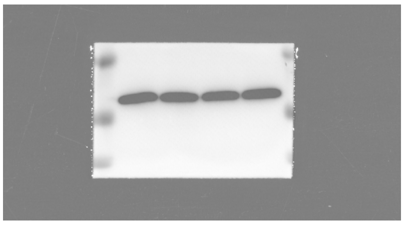

Supplementary Figure 8A

Lamin A/C

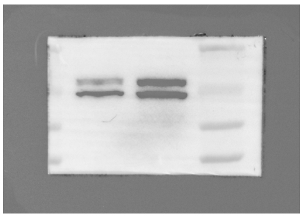

p-Lamin A/C

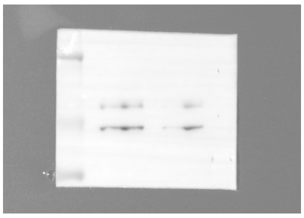

Lamin B1

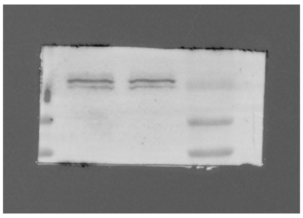

GAPDH

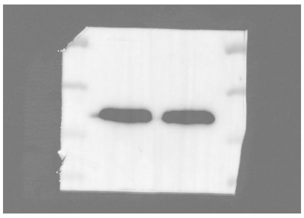

Supplementary Figure 8C

IB β-catenin

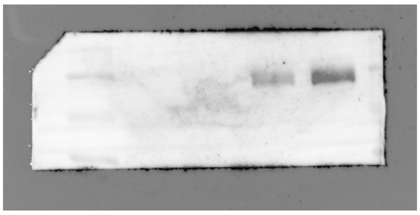

IB Lamin A/C

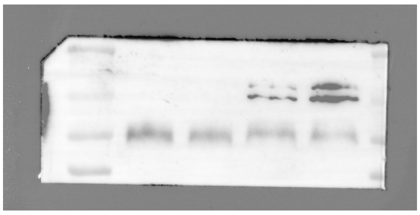

Input β-catenin

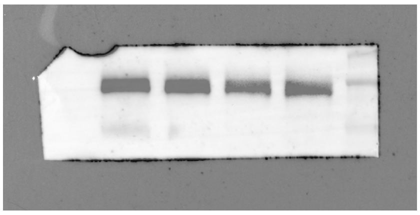

Input Lamin A/C

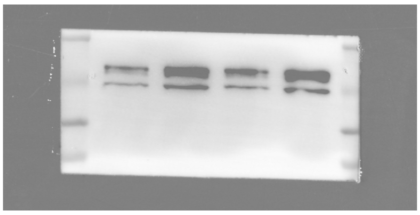

GAPDH

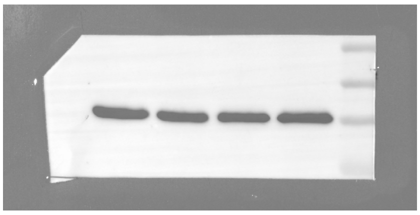

Supplementary Figure 8E

IB β-catenin

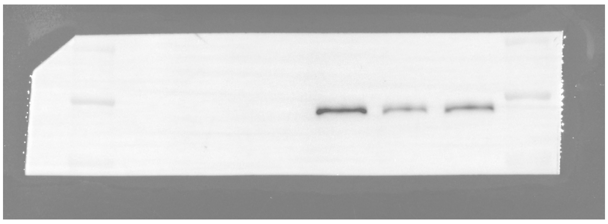

IB Lamin A/C

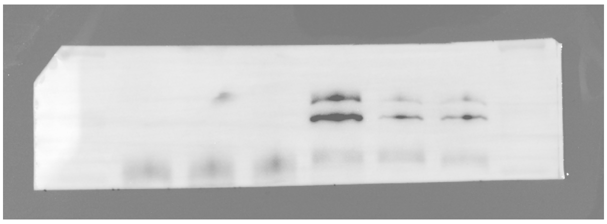

Input β-catenin

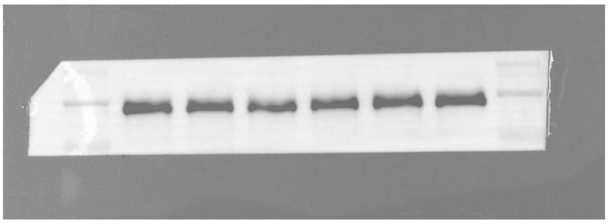

Input Lamin A/C

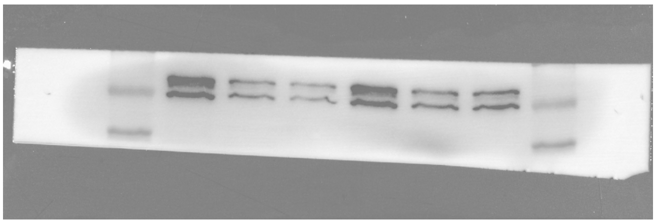

GAPDH

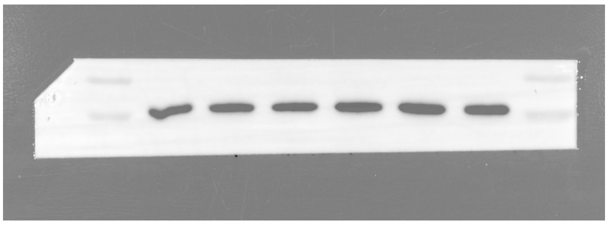

Supplementary Figure 8G

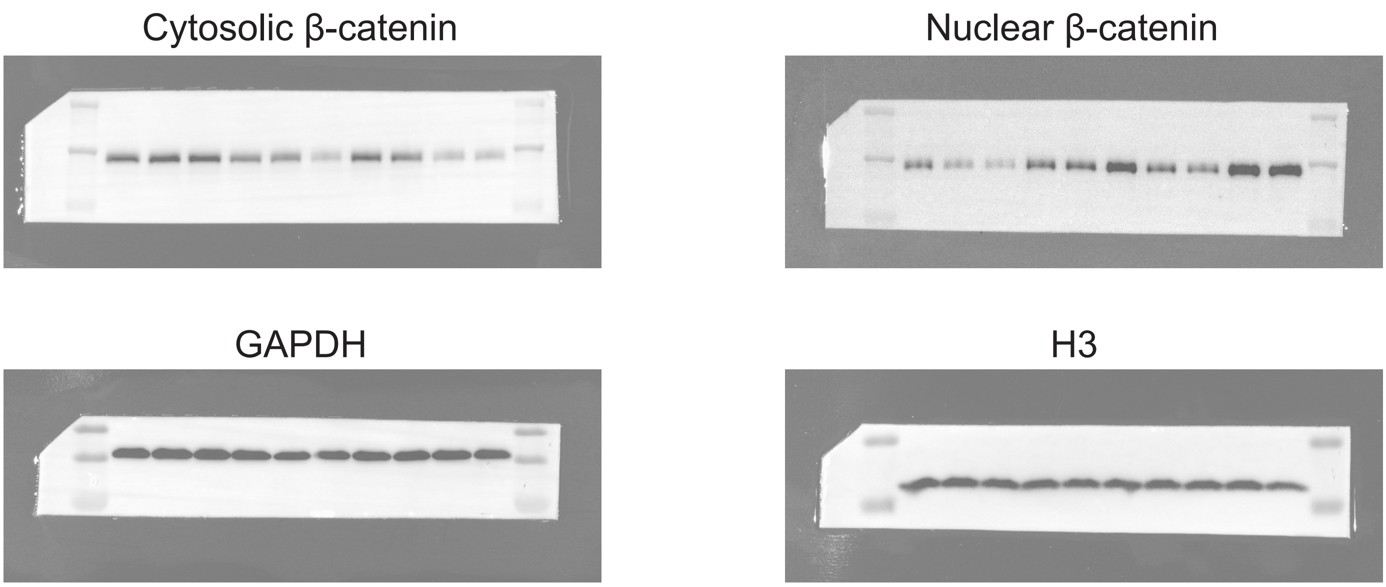

Supplementary Figure 9A

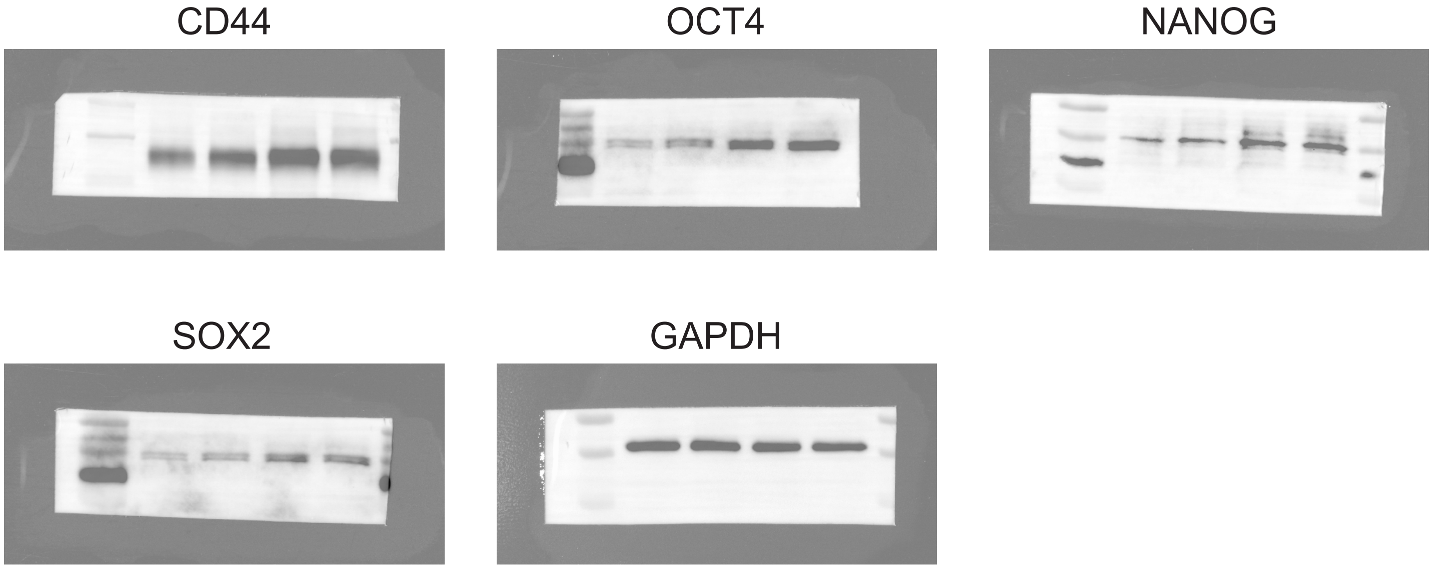

Supplementary Figure 9D

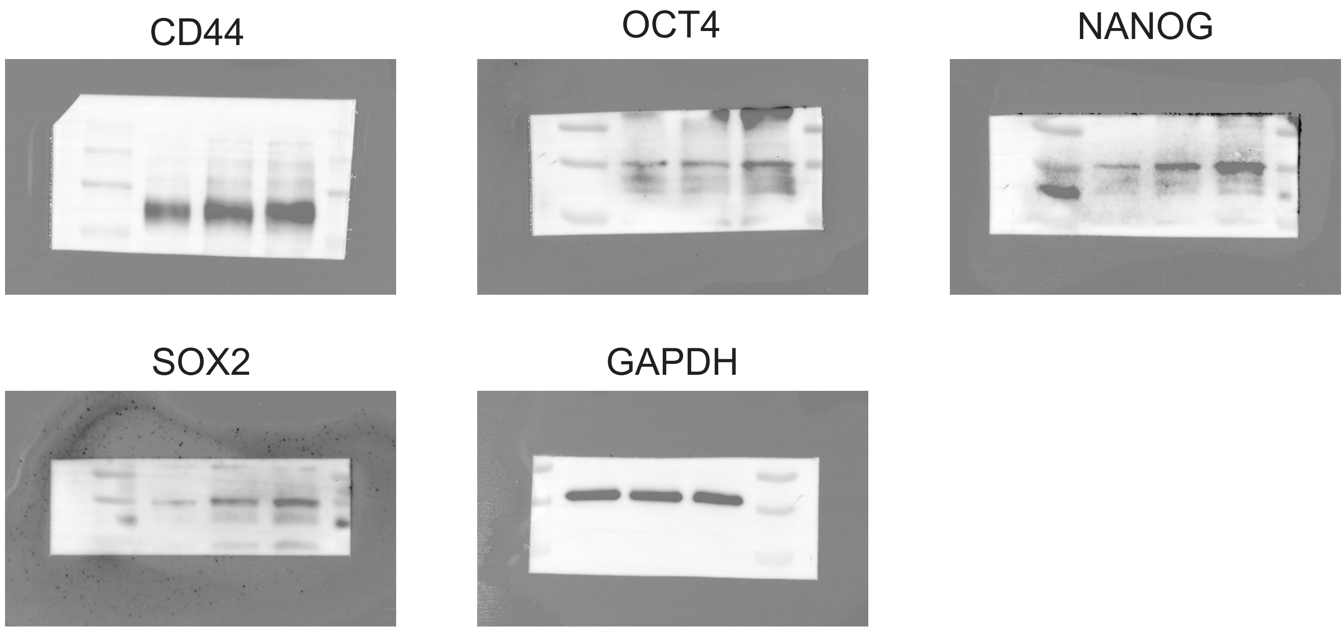

Supplement: Supplementary file 13 — Uncropped western blots [file 41419_2025_8222_MOESM13_ESM.pdf]
